# Surface potential-adjusted surface states in 3D topological photonic crystals

**DOI:** 10.1038/s41598-024-56894-6

**Published:** 2024-03-26

**Authors:** Haedong Park, Sang Soon Oh, Seungwoo Lee

**Affiliations:** 1 KU-KIST Graduate School of Converging Science and Technology, Korea University, Seoul, 02841 Republic of Korea; 2 School of Physics and Astronomy, Cardiff University, Cardiff, CF24 3AA UK; 3 Department of Biomicrosystem Technology, Korea University, Seoul, 02841 Republic of Korea; 4 Department of Integrative Energy Engineering and KU Photonics Center, Korea University, Seoul, 02841 Republic of Korea; 5 Center for Opto-Electronic Materials and Devices, Post-Silicon Semiconductor Institute, Korea Institute of Science and Technology (KIST), Seoul, 02792 Republic of Korea

**Keywords:** Metamaterials, Photonic crystals, Magneto-optics, Photonic devices

## Abstract

Surface potential in a topological matter could unprecedentedly localize the waves. However, this surface potential is yet to be exploited in topological photonic systems. Here, we demonstrate that photonic surface states can be induced and controlled by the surface potential in a dielectric double gyroid (DG) photonic crystal. The basis translation in a unit cell enables tuning of the surface potential, which in turn regulates the degree of wave localization. The gradual modulation of DG photonic crystals enables the generation of a pseudomagnetic field. Overall, this study shows the interplay between surface potential and pseudomagnetic field regarding the surface states. The physical consequences outlined herein not only widen the scope of surface states in 3D photonic crystals but also highlight the importance of surface treatments in a photonic system.

## Introduction

The discovery of topological insulators opened exciting new realms of physics^1,2^, and extensive efforts have been made to understand topological physics over the last decade. At this field’s core, it is important to note zero-dimensional degeneracies such as Dirac^3–9^ or Weyl points^10–19^ and one-dimensional degeneracies such as nodal lines^20–28^. These band degeneracies have been theoretically or experimentally realized using metals^21,29^, semimetals^30–36^, phononic crystals^37–39^, electrical circuits^40^, and photonic crystals^10,11,18,41–49^. Based on the definition of topological insulators, many studies have proposed several boundary states, such as one-way surface/edge states^39,50–53^, drumhead surface states^40,54–57^, and Fermi arcs^47,48,58–61^.

Beyond them, one can adjust the boundary state with surface/edge potential^36,62–68^ and pseudomagnetic field^13,37,69^. A realistic system always has surfaces/edges, and the surfaces/edges act benefit or obstacle for the theoretical predictions to be realized by experimental performances. In condensed matters, the surface/edge potential^36,62–65^ arises from the passivation on the surface/edge of a given material^66–68^. Such potentials have enabled the edge state adjustment, similar effect to the application of an external field, and imposing a perturbation. The surface/edge potential^36,62–64^ has been successfully implemented in two-dimensional (2D) materials including graphene^66^, boron nitride^67^, and semimetals;^36,65,68^ consequently, the excitation and manipulation of surface/edge states came to the fore. However, such driving and using surface potential are yet to be much exploited in 3D topological photonic crystals. Furthermore, there are relatively few studies about the interplay between the perturbations by surface/edge and bulk of a finite sized crystal.

Meanwhile, the pseudomagnetic field is a virtual effective field stemming from a spatial reconfiguration of the crystal lattice without a real magnetic field^70–73^. Like a real magnetic field enabling the quantum Hall effect^74,75^, the pseudomagnetic field was found to be a gold vista for topologically nontrivial surface/edge states in semimetals^76–80^, photonic crystals^81–84^ and phononic crystals^37,71,72,85^. Nevertheless, the surface state’s profiles along the zeroth Landau level on a 3D Weyl photonic crystal, driven by the pseudomagnetic field, were out of reach.

Here, we demonstrate photonic surface waves that arise from the interplay between surface potential and pseudomagnetic field in photonic systems. First, we investigate the effect of the surface potential in a system governed by the Weyl equation with a pseudomagnetic field. The pseudomagnetic field is switched on by the Weyl points that vary with the unit cell positions along the boundary-boundary direction in the system. Then, we apply the effective Hamiltonian description to a photonic system based on double gyroid (DG) photonic crystals^17^. The DG photonic crystals are found to exhibit Weyl points due to the geometrical perturbation, which appears as a defect-like shape. To realize a pseudomagnetic field, we constitute a photonic array of the DG unit cells with a spatial gradient of the perturbation, i.e., the degree of the defect varies with the position. We then compute quantized Landau levels and eigenstates along the zeroth Landau level to quantitate the asymmetric localization of photonic waves on the surfaces. This observed asymmetry in wave localization evidences the existence of the surface potential. Then, we tune the translation of the basis of the unit cell to tame the surface termination. This tuning exquisitely controls the surface potential so that the degree of wave localization varies with respect to the tuning. Finally, we implement such results to the evasion behaviors of a photonic wave to observe the interplay between the surface potential and pseudomagnetic field.

## Results

### The attraction of waves by the surface potential in the Weyl system

**Figure 1:**
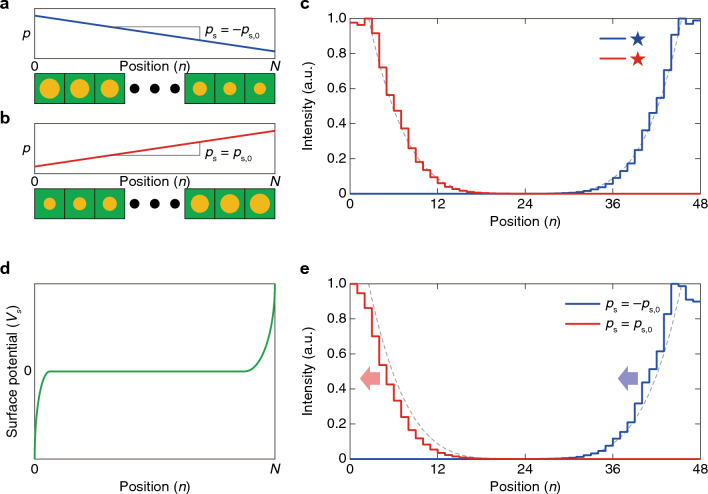
Effects of surface potential on the wave localization by pseudomagnetic fields. (**a**, **b**) Schematic illustrations of two systems with opposite ${p}_{s}$. The systems are finite along ${x}_{3}$-direction (the horizontal direction) and periodic along ${x}_{1}$- and ${x}_{2}$-direction. In each panel, the varying $p$ is schematically represented as the size of yellow circles. Both systems are described by Eq. (1). (**c**) Comparisons of their eigenstates’ distributions along ${x}_{3}$-direction, exhibiting the mutual symmetric dispersions. The eigenstates are calculated by Eq. (1). (**d**) Schematic plot of ${V}_{s}$, the scalar coefficient of the surface potential. (**e**) Comparisons of the eigenstates’ distributions of (**a** and **b**) when ${V}_{s}$ is applied. Here, the eigenstates are calculated by Eq. (4). In (**c** and **e**) gray dotted lines are the symmetric curves with respect to the center for comparisons of the red and blue plots.

First, let us describe the pseudomagnetic field’s effects using the Weyl Hamiltonian. For this, we consider a system periodic along the ${x}_{1}$- and ${x}_{2}$-directions for an orthogonal coordinate system. The system consists of $N$ unit cells along the ${x}_{3}$-direction (the horizontal rightward direction in Fig. 1a-b), and surface boundaries are parallel to the ${x}_{1}$- and ${x}_{2}$-directions. We assume that this system is governed by

1 $${\varvec{\upsigma}}\cdot \left(-i\nabla -{\mathbf{k}}_{\text{w}}\right)\psi =i{\sigma }_{0}\frac{\partial \psi }{\partial t}$$

that describes a Weyl point at ${\mathbf{k}}_{\text{w}}$, where ${\sigma }_{0}$ is the 2 × 2 identity matrix, and ${\sigma }_{i}$ ($i=1, 2, 3$) are the Pauli matrices. We decompose the Weyl point’s location ${\mathbf{k}}_{\text{w}}$ into ${\mathbf{k}}_{\text{w}}={\mathbf{k}}_{{\text{w}},0}+{\mathbf{A}}_{\text{w}}^{n}$, as introduced in Refs. ^13,37,71,85^. ${\mathbf{k}}_{{\text{w}},0}$ is a constant while ${\mathbf{A}}_{\text{w}}^{n}$ depends on the unit cell’s index $n$. We assume that ${\mathbf{A}}_{\text{w}}^{n}$ linearly varies with a specific value $p$ (henceforth, this value is referred to as perturbation strength) which also linearly varies with $n$:

2 $${\mathbf{A}}_{\text{w}}^{n}\propto p\left(n\right)={p}_{s}n+{\text{const.}},$$

where ${p}_{s}$ is a proportional constant (refer to Fig. 1a,b). Then, the pseudomagnetic field can be written as $\mathbf{B}=\nabla \times {\mathbf{A}}_{\text{w}}^{n}$, which is proportional to ${p}_{s}$.

Let us suppose two systems with different signs of slopes, i.e., one system described by ${p}_{s}=-{p}_{s,0}$ (Fig. 1a) and the other described by ${p}_{s}=+{p}_{s,0}$ (Fig. 1b) where ${p}_{s,0}$ is positive. Their $p\left(n\right)$ are schematically illustrated as the size of yellow circles in Fig. 1a,b. The pseudomagnetic fields $\mathbf{B}$ for these two are in the same magnitudes and in opposite directions. Their wave localization of the zeroth Landau level at a specific point in the momentum space shows mutually symmetric characteristics for these opposite fields, as shown in Fig. 1c.

Now, let us implement a surface potential $V$ as follows:

3 $$V={V}_{s}{\sigma }_{3} ,$$

where ${V}_{s}$ is nonzero only around the boundaries^36,62,65–67^, like Fig. 1d. When we include the surface potential term with ${V}_{s}$, further detailed in Methods, Eq. (1) is rewritten as:

4 $$\left\{{\varvec{\upsigma}}\cdot \left(-i\nabla -{\mathbf{k}}_{\text{w}}\right)+V\right\}\psi =i{\sigma }_{0}\frac{\partial \psi }{\partial t} .$$

By applying the same $V$ for the two systems in Fig. 1a,b, we can observe mutually asymmetric localization, as shown in Fig. 1e. The two localized states commonly show a slight shift in the left direction, implying that the surface potential attracts them along that direction.

Derivation of a pseudomagnetic field with Eqs. (1) and (2) and the relevant data in Fig. 1a,c are not new as they already have been introduced in several studies^13,37,71,85^. The same explanation is applied to the concept of the surface potential^36,62,65–67^. However, to our knowledge, adopting a surface potential into the pseudomagnetic field has not been tried. Thus, the next several sections are discussions about how to interplay them.

### Applying surface potential in a finite-sized array

**Figure 2:**
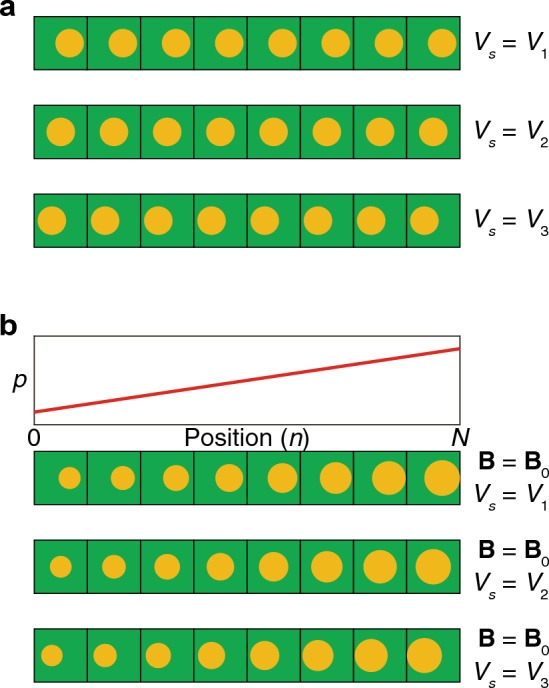
Schematics on the realization of surface potential. (**a**) Inducing different surface potentials by different surface terminations. The surface terminations are adjusted by the basis’s positions in a unit cell. (**b**) Adding pseudomagnetic field on the systems in (**a**). Generating surface localization of a photonic wave is performed by the pseudomagnetic field, and the degree of localization depends on the surface potential.

Although there can be several methods to drive a surface potential into a photonic crystal, we herein adjust the surface terminations in a finite-sized array. In a fully periodic crystal, the fields or eigenvectors of a propagating mode are invariant under discrete translation, not depending on a specific position of the basis in a unit cell. On the contrary, a finite-sized array has terminations at the boundaries. A propagating wave profile depends on the terminations according to the variations of the basis’s positions in the unit cells^86^, as shown in Fig. 2a. Thus, the effect of surface potential can be quantitated with respect to adjusting the surface terminations.

Here, we remark on the followings: (i) Eqs. (1) to (4) do not consider a detailed geometry in each unit cell. Thus, observation of the relation between the surface termination and surface potential should be phenomenologically carried out using a real array structure. (ii) A photonic band structure for a finite-sized array displays projected bands, called folding of bands. Without the pseudomagnetic field, the zeroth Landau level adheres to the bulk bands so that surface states cannot be obtained. Thus, we should use the pseudomagnetic field, as shown in Fig. 2b. Our study shows the interplay between the surface potential and pseudomagnetic field; the pseudomagnetic field gives the surface localization of a photonic wave, and the surface potential tunes the degree of the localization.

To build the array in Fig. 2b and to induce both the surface potential and pseudomagnetic field, we use a DG photonic crystal that exhibits Weyl points^17^. We adjust the surface terminations by tuning the translation of the basis along the normal direction to the boundaries.

### Double gyroid photonic array exhibiting pseudomagnetic field

**Figure 3:**
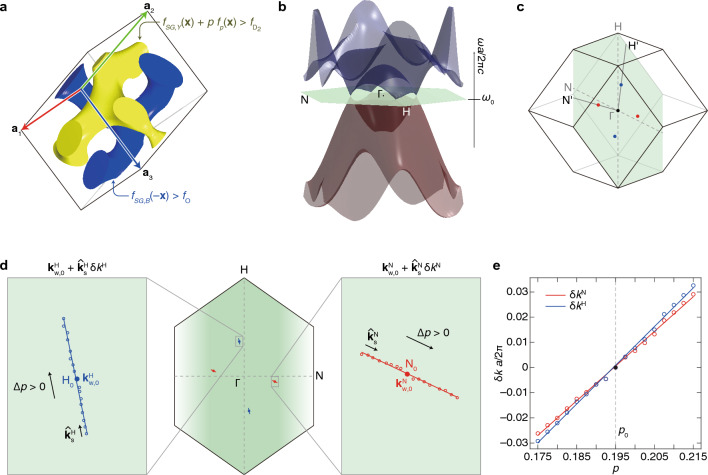
Design of 3D photonic crystal for pseudomagnetic field. (**a**) A DG photonic crystal. The SGs are defined by $\mathbf{x}$ that satisfies the inequalities denoted in the figure. The yellow SG’s inequality has an additional term $p{f}_{p}\left(\mathbf{x}\right)$ to impose a defect-like shape on this structure (see the bottom-right arm). (﻿**b**) Simulated photonic band structure exhibiting four Weyl points, the band degeneracies. ﻿(**c**) Weyl points marked in the three-dimensional first Brillouin zone. The four Weyl points in (**b**) are marked on the green plane. (**d**) Simulation result of Weyl points’ movements on the plane with the perturbation strength $p$. Weyl points in ﻿(**c**) are also marked here with red and blue solid points and denoted by ${{\text{N}}}_{0}$ and ${{\text{H}}}_{0}$, respectively. ﻿(**e**) Deviations of the Weyl points’ movements with $p$ from the positions of ${{\text{N}}}_{0}$ and ${{\text{H}}}_{0}$, shown in the right and left enlargements in ﻿(**d**), respectively.

To drive the surface potential by the surface termination, we use a photonic array that exhibits a pseudomagnetic field by ${\mathbf{k}}_{{\text{w}}}={\mathbf{k}}_{{\text{w}},0}+{\mathbf{A}}_{\text{w}}^{n}$ in Eq. (4). We consider the DG, as shown in Fig. 3a. The yellow and blue single gyroids (SGs) are given by a set of $\mathbf{x}=\left[{x}_{1},{x}_{2},{x}_{3}\right]$ such that ${f}_{SG,Y}\left(\mathbf{x}\right)+p{f}_{p}\left(\mathbf{x}\right)>{f}_{{D}_{2}}>0$ and ${f}_{SG,B}\left(-\mathbf{x}\right)>{f}_{O}>0$, respectively, where ${f}_{{D}_{2}}$ and ${f}_{O}$ are the level-set values that determine the volume fraction of each SG. The yellow SG has selectively the perturbation term $p{f}_{p}\left(\mathbf{x}\right)$ to break the inversion symmetry and to generate Weyl points in momentum space. Due to this perturbation, the yellow SG exhibits a defect-like narrow region on the arm passing the ${\mathbf{a}}_{1}{\mathbf{a}}_{2}$ surface, as shown in Fig. 3a. The higher perturbation strength $p$ induces the deeper defect-like shape; this corresponds to the yellow circle’s size in Fig. 1a,b and Fig. 2. (See Methods for the detailed explanations about the DG crystal.) When this DG photonic crystal is periodic along all three lattice vector directions, it can exhibit four Weyl points, as shown in Fig. 3b,c. The Weyl points ${{\text{H}}}_{0}$ and ${{\text{N}}}_{0}$ (marked with blue and red solid points in Fig. 3c, respectively) have positive and negative topological charges, respectively.

Varying $p$ of the DG shifts the positions of the Weyl points, as shown in Fig. 3d. We denote the moved positions of the Weyl points ${{\text{N}}}_{0}$ and ${{\text{H}}}_{0}$ in momentum space by ${\mathbf{k}}_{{\text{w}}}^{{\text{N}}}$ and ${\mathbf{k}}_{{\text{w}}}^{{\text{H}}}$, respectively. Increasing $p$ makes the Weyl points shift away from Γ-point on the single plane ($\left(001\right)$-plane). For the narrow range of $p$ around the central value ${p}_{0}$, the traces of all Weyl points exhibit linear shapes so that we can write their positions as ${\mathbf{k}}_{{\text{w}}}^{{\text{H}}}={\mathbf{k}}_{{\text{w}},0}^{{\text{H}}}+{\widehat{\mathbf{k}}}_{{\text{s}}}^{{\text{H}}}\updelta {k}^{{\text{H}}}$ and ${\mathbf{k}}_{{\text{w}}}^{{\text{N}}}={\mathbf{k}}_{{\text{w}},0}^{{\text{N}}}+{\widehat{\mathbf{k}}}_{{\text{s}}}^{{\text{N}}}\updelta {k}^{{\text{N}}}$ where ${\widehat{\mathbf{k}}}_{{\text{s}}}^{{\text{H}}}$ and ${\widehat{\mathbf{k}}}_{{\text{s}}}^{{\text{N}}}$ are overall directions of the traces (see Fig. 3d). The deviations of shifted Weyl points, $\updelta {k}^{{\text{N}}}$ and $\updelta {k}^{{\text{H}}}$, also show linear relations with $p$, as shown in Fig. 3e.

**Figure 4:**
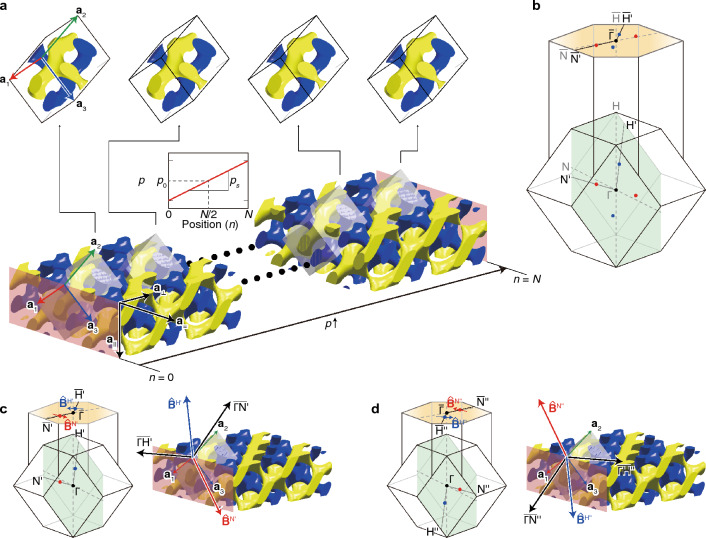
Design of a photonic array and resulting pseudomagnetic fields. (**a**) Schematic geometry which consists of $N$ cells with spatially linearly varying $p$ along ${\mathbf{a}}_{\perp }$-direction. This gradient is imposed only on the yellow structure. (**b**) Bulk and surface Brillouin zones. (**c**, **d**) Direction vectors of the pseudomagnetic field around each Weyl point by the above design, denoted by ${\widehat{\mathbf{B}}}^{{{\text{N}}}^{\mathrm{^{\prime}}}}$, ${\widehat{\mathbf{B}}}^{{{\text{H}}}^{\mathrm{^{\prime}}}}$, ${\widehat{\mathbf{B}}}^{{{\text{N}}}^{\mathrm{^{\prime}}\mathrm{^{\prime}}}}$, and ${\widehat{\mathbf{B}}}^{{{\text{H}}}^{\mathrm{^{\prime}}\mathrm{^{\prime}}}}$, respectively. These are calculated from the traces around ${{\text{N}}}_{0}$, ${{\text{H}}}_{0}$, $-{{\text{N}}}_{0}$, and $-{{\text{H}}}_{0}$ marked in Fig. 3d, respectively.

To generate the pseudomagnetic field, we design a photonic array made of DGs whose $p$ linearly varies with respect to the position in the array^13,71,72,87,88^. First, we assume a DG-array that consists of DGs with $N$ unit cells along the ${\mathbf{a}}_{\perp }$-direction between two boundaries, as shown in Fig. 4a. The array is periodic along the ${\mathbf{a}}_{=}$- and ${\mathbf{a}}_{\parallel }$-directions, but does not preserve the translational symmetry along the ${\mathbf{a}}_{\perp }$-direction. Next, we apply the non-uniform geometry on this array using the perturbation strength $p$ that linearly varies along the ${\mathbf{a}}_{\perp }$:

5 $$p={p}_{s}\left\{{x}_{\perp }-{d}_{0}\right\}+{p}_{0} ,$$

like the inset linear plot in Fig. 4a. Here, ${x}_{\perp }$ is a coordinate along the ${\mathbf{a}}_{\perp }$, ${d}_{0}$ is a distance between the planes at $n=0$, and $n=N/2$, and ${p}_{0}$ is a central value of $p\left(\mathbf{x}\right)$. Thus, the location of the Weyl point varies with the ${\mathbf{a}}_{\perp }$-directional coordinate. From the information in Fig. 4a,b, the pseudomagnetic field has the form $\mathbf{B}={\nabla }_{\mathbf{x}}\times \mathbf{A}={p}_{s}\left({B}_{=}{\widehat{\mathbf{a}}}_{=}+{B}_{\parallel }{\widehat{\mathbf{a}}}_{\parallel }\right)$, which are parallel to the array boundaries (see the orange planes in Fig. 4c,d). As a result, the waves can be localized at the boundaries (see Methods for detailed explanations about the array and pseudomagnetic field calculations).

### Surface potential by surface termination and resulting photonic wave localization

Plugging the design in the previous section to the array of $N=48$ primitive cells allows us to generate Landau spectra. The detailed explanations of the Landau levels related to the pseudomagnetic field are in Sect. 1, Supplementary Information.

**Figure 5:**
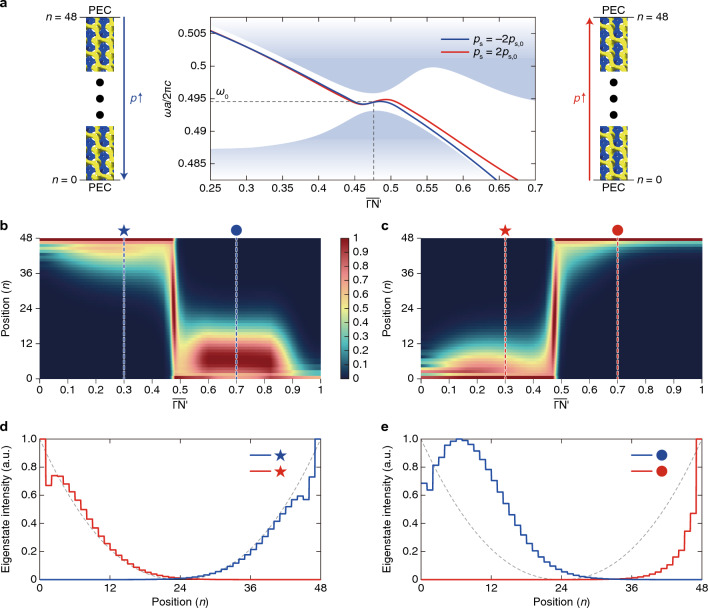
Photonic wave localization and evidence of surface potential. (**a**) Comparison of Landau levels by the opposite perturbation fields. The left and right cases correspond to ${p}_{s}=-2{p}_{s,0}$ and ${p}_{s}=2{p}_{s,0}$, respectively. (**b**, **c**) Normalized eigenstates respectively along the blue- and red-colored Landau levels shown in (**a**) to see surface states localized on the boundaries. (**d**, **e**) Comparisons of the wave localization along the vertical lines in (**b** and **c**). Data with the same symbols are overlapped in the same plot. Gray dotted lines are the symmetric curves with respect to $n=24$ for comparisons of the red and blue plots. All the plots are simulation results.

Here, we extract and compare the zeroth Landau levels by two arrays with ${p}_{s}=-2{p}_{s,0}$ and ${p}_{s}=+2{p}_{s,0}$, as illustrated in Fig. 5a. Although the two arrays have different internal geometries due to the different ${p}_{s}$ values, their overall translation status are identical, i.e., they generally use the formulae denoted in Fig. 3a. Thus, we consider that their surface terminations are identical. The photonic band structure in Fig. 5a shows that the resulting zeroth Landau levels accessible from the two arrays are not equal. Furthermore, the eigenstate intensity distributions for the two cases reveal mutual asymmetric (Fig. 5b,c). The distributions in Fig. 5d,e are biased toward the $n=48$ from the symmetric curve (the gray dotted lines). From the fact that the results in Fig. 5d,e exhibit the same tendency as with Fig. 1e, we can conclude that there exists a surface potential in these arrays.

**Figure 6:**
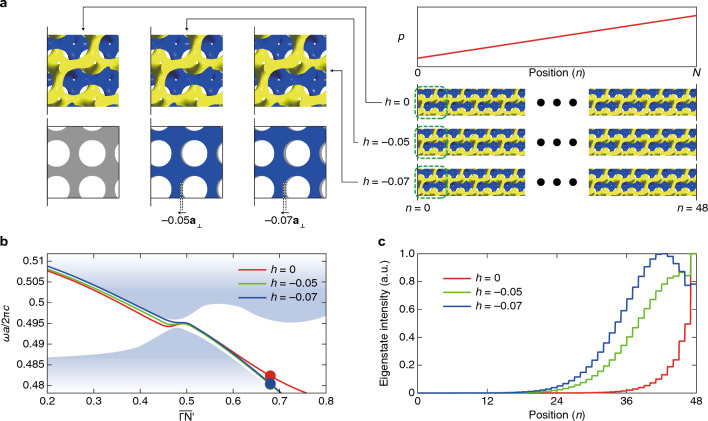
Adjustment of surface potential by surface termination. (**a**), Schematic illustration of three surface terminations. Observation of the surface potentials is carried out by adjusting the basis’s positions in each unit cell. The upper left three structures are enlargements of the bottom right arrays. The lower left three figures are respectively the schematics of the upper left structures, and they clearly show the translation of the structures along ${\mathbf{a}}_{\perp }$-direction. The right upper plot indicates that the perturbation $p$ versus the position $n$ is the same for all three cases. (**b**, **c**) Simulation results: zeroth Landau levels (**b**) and eigenstate intensities (**c**) at the point marked in (**b)** for three different surface terminations tuned by translation $h$ along the ${\mathbf{a}}_{\perp }$-direction when $N=48$ and ${p}_{s}=2{p}_{s,0}$ are used.

Now, we replace $\mathbf{x}$ as $\mathbf{x}-h{\mathbf{a}}_{\perp }$ to apply overall translation $h$ of the DG by $h\left|{\mathbf{a}}_{\perp }\right|$ along the ${\mathbf{a}}_{\perp }$-direction to verify the surface termination’s effect, as illustrated in Fig. 6a. Note that the profile of $p$ does not move (see the upper right plot in Fig. 6a). For a specific point on the zeroth Landau levels (marked in Fig. 6b), we calculate the field intensity for several $h$ values, as shown in Fig. 6c. The discussions so far are about that the surface potential can adjust the degree of localization of surface states generated by the pseudomagnetic field. The results in Fig. 6c indicate that the localization degree can be adjusted by the surface termination. The zeroth Landau level in the photonic band structure also can be moved to another position by changing $h$. Due to the relatively small $h$, the overall configuration of the zeroth Landau level and the surrounding bulk bands remains almost intact, as shown in Fig. 6b.

### Evasion behavior of photonic wave

We embody the evasion behavior of a photonic wave in a DG structure using oppositely graded $p$. Adjusting the propagation path of a photonic or phononic wave has been an interest in photonics or wave mechanics. These have been proved using materials with negative-refractive indices^16,89–91^ or Dirac/Weyl crystals exhibiting one-way propagations of waves^3,12,13,44,92^. Such proofs are related to invisibility-cloaking. Here, we perform the behavior by pulling a photonic wave that was originally propagating on one boundary toward the opposite boundary.

**Figure 7:**
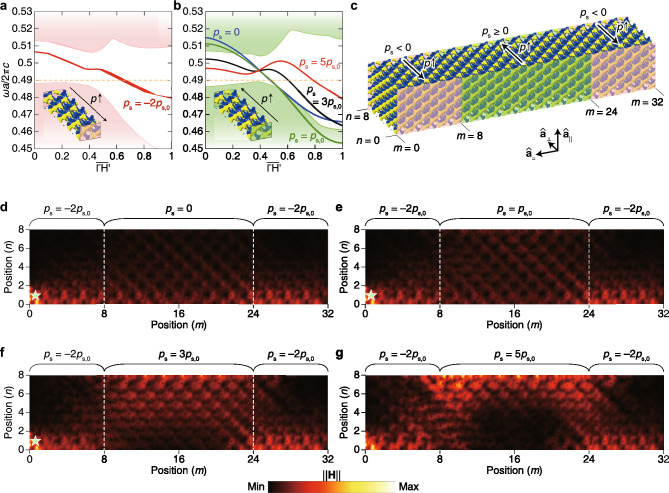
Evasion behavior of photonic waves using heterogeneous blocks. (**a**, **b**) Zeroth Landau levels by the 8-block system with several perturbation fields, where ${p}_{s,0}=7.0711\times {10}^{-3}{a}^{-1}$. The perturbation fields used in (**a**, **b**) are opposite. (**c**) Heterogeneous DG system. The PEC is applied to the pink and green boundaries. In pink and green colored bounded blocks, the perturbation fields’ directions are opposite as marked. The system is periodic only along ${\widehat{\mathbf{a}}}_{\parallel }$-direction. (**d**–**g**) Evasion behavior of photonic waves with several ${p}_{s}>0$ values of the central blocks with fixing ${p}_{s}<0$ values of the blocks around both ends. Incident points are marked as the star symbols.

DG arrays that consist of 8 cells with ${p}_{s}=-2{p}_{s,0}$, $0$, ${p}_{s,0}$, $3{p}_{s,0}$, and $5{p}_{s,0}$ are prepared, based on the array in Fig. 4a, (${p}_{s,0}>0$). (The number of cells is counted based on the body-centered cubic primitive cell.) The reason for using a smaller number of cells than in the previous case is to broaden intervals between Landau levels. We classify these arrays into groups with ${p}_{s}=-2{p}_{s,0}$ and the others with ${p}_{s}\ge 0$. The directions of ${\nabla }_{\mathbf{x}}p$ for these two groups are opposite to each other (see the inset of Fig. 7a,b). The band structures for ${p}_{s}<0$ and ${p}_{s}\ge 0$ (plotted in Fig. 7a,b, respectively) exhibit Landau levels along $\Gamma {{\text{H}}}^{\mathrm{^{\prime}}}$-direction. From these plots, we consider $\omega a/2\pi c=0.49$ as an optimized frequency for the evasion behavior because this frequency between the bulk bands and all zeroth Landau levels commonly meets this frequency only once. We then constitute a DG array, as shown in Fig. 7c, using the 8-cells arrays depicted in the inset of Fig. 7a,b. Each system consists of 8 and 32 cells along ${\mathbf{a}}_{\perp }$- and ${\mathbf{a}}_{=}$-directions, respectively, and it is assumed to be infinitely periodic along ${\widehat{\mathbf{a}}}_{\parallel }$-direction. The blocks $m=\left[0, 8\right]$ and $m=\left[24, 32\right]$ use negative-valued ${p}_{s}$, and the central region between the two sections has positive-valued ${p}_{s}$.

The DG array reveals the evasion behavior of photonic waves. At $\omega a/2\pi c=0.49$, a photonic wave is localized in the section of ${p}_{s}=-2{p}_{s,0}$ around the boundary at $n=0$. We then input an incident photonic wave around the localization region, as marked by the star symbols in Fig. 7d–g. If the blocks $m=\left[8, 24\right]$ are the same as the other blocks, the localization would continue in the central region. We simulate here the situations that the central region’s ${p}_{s}$ is respectively $0$, ${p}_{s,0}$, $3{p}_{s,0}$, and $5{p}_{s,0}$. The results show the gradual attraction of the photonic waves onto the boundary at $n=8$ with increasing ${p}_{s}$ (see Fig. 7d–g).

In addition, we observe the attraction of waves towards the $n=0$ boundaries. In the blocks $m=\left[0, 8\right]$ and $m=\left[24, 32\right]$, the waves are localized on the boundary. On the contrary, the localized waves in the blocks $m=\left[8, 24\right]$ exhibit biased behaviors even when the central blocks’ ${p}_{s}$ is $3{p}_{s,0}$ or $5{p}_{s,0}$. This coincides to the mutual asymmetric distributions of the waves shown in Fig. 5e, and we see here the existence of a surface potential in the blocks. Then, we can conclude that this arises from surface potential and surface termination. If we adjust the surface termination, we may observe the bias towards the opposite direction. All these show the interplay between surface potential and pseudomagnetic field.

## Discussion

We have demonstrated the asymmetric localization of photonic waves via an interplay between surface potential and pseudomagnetic field using a DG photonic crystal. The pseudomagnetic field has induced the surface states of the photonic waves and the surface potential adjusted the degree of localization. The pseudomagnetic field was formed by the graded location of Weyl points, and the surface potential was applied using surface termination by tuning unit cells’ basis translation. We have observed the shift of the surface states as a result of the surface potential. In fact, the interplay by the surface potential and pseudomagnetic field generates natural and predictable results so that this might be underestimated. However, the various methods of applying a surface potential (even though they have been utilized in metals, semimetals, or acoustics) can be interplayed with a pseudomagnetic field in photonics in the near future. Thus, we expect that our current study will be a starting point of these directions.

In the case of graphene^66^, boron nitride^67^, and semimetals^36,65,68^, surface potentials can be applied using materials’ surface/edge passivation. Although this study used surface termination by crystal’s basis translation, we believe that there could be several types of photonic passivation, for example, doping with a thin material such as a dielectric sheet, imposing perfectly magnetic conductor boundary condition, cutting position of the crystal, or attaching a band-gap material. Then, this study and the follow-up studies will open the various possibility of using topological photonic crystals. Meanwhile, there is no example of the detailed analysis of the surface states along the zeroth Landau level by the Hall effect and pseudomagnetic field in three-dimensional Weyl photonic crystals. Therefore, this study will fill this gap thereby this study will positively affect the other studies on the three-dimensional quantum (spin) Hall effect^93–98^.

## Materials and methods

### Eigenstates localization by Weyl Hamiltonian

**Figure 8:**
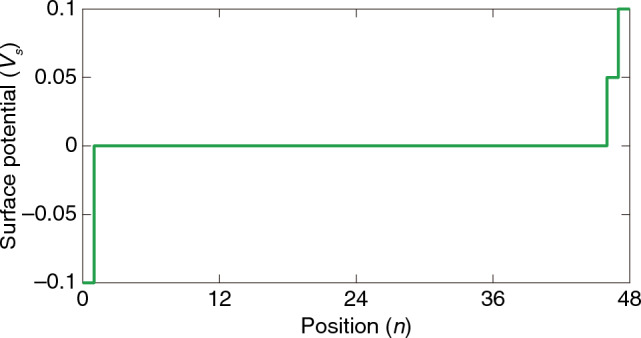
Plot of the surface potential’s scalar coefficient ${V}_{s}$**.** This is the discretized function from Fig. 1d. Plots in Fig. 1c,e were obtained using this ${V}_{s}$.

We apply Eq. (1) or (4) to the finite system illustrated in Fig. 1a-b to observe the wave localization like Fig. 1c or e. Instead of deriving the Landau level analytically using ladder operators^33,99^, we use a traveling wave solution $\psi \left(\mathbf{x},t\right)=u{e}^{i\left(\mathbf{k}\cdot \mathbf{x}-\omega t\right)}$ to consider the finite array. The traveling wave solution can be rewritten as the product of a state ${u}_{n}$ that depends on only $n$ and an exponent ${e}^{i\left({k}_{1}{x}_{1}+{k}_{2}{x}_{2}-\omega t\right)}$ that depends on only other variables: $\psi \left(\mathbf{x},t\right)={u}_{n}{e}^{i\left({k}_{1}{x}_{1}+{k}_{2}{x}_{2}-\omega t\right)}$. The surface localization is obtained by substituting this into Eq. (1) or (4). To consider the surface potential in Eq. (3), we used the plot in Fig. 8 as ${V}_{s}$. Detailed derivations, explanations, and additional results related to Fig. 1 are given in Sect. 2, Supplementary Information.

### Preparations of DG photonic crystal

In the following, we give detailed explanations of the DG photonic crystal and array. In this study, we consider the DG, reported in ref.^17^. The body-centered cubic primitive cell of this structure is defined by the lattice vectors ${\mathbf{a}}_{i}=a/2\left[\mathrm{1,1},1\right]-a{\widehat{\mathbf{x}}}_{i}$, where $\left[{\widehat{\mathbf{x}}}_{1}{\widehat{\mathbf{x}}}_{2}{\widehat{\mathbf{x}}}_{3}\right]=I$. The mathematical formulae of ${f}_{SG,Y}\left(\mathbf{x}\right)$, ${f}_{p}\left(\mathbf{x}\right)$, and ${f}_{SG,B}\left(\mathbf{x}\right)$ denoted in Fig. 3a are ${f}_{SG,Y}\left(\mathbf{x}\right)={\text{sin}}{X}_{1}{\text{cos}}{X}_{2}+{\text{sin}}{X}_{2}{\text{cos}}{X}_{3}+{\text{sin}}{X}_{3}{\text{cos}}{X}_{1}$, ${f}_{p}\left(\mathbf{x}\right)={\text{sin}}\left({X}_{1}+{X}_{2}\right)$, and ${f}_{SG,B}\left(\mathbf{x}\right)={\text{sin}}{\widetilde{X}}_{1}{\text{cos}}{\widetilde{X}}_{2}+{\text{sin}}{\widetilde{X}}_{2}{\text{cos}}{\widetilde{X}}_{3}+{\text{sin}}{\widetilde{X}}_{3}{\text{cos}}{\widetilde{X}}_{1}$, respectively. Here, the local coordinates are given by $\mathbf{X}=\left[{X}_{1},{X}_{2},{X}_{3}\right]=\left(2\pi /a\right)\left(\mathbf{x}-{\mathbf{a}}_{s}s\right)$ and $\widetilde{\mathbf{X}}=\left[{\widetilde{X}}_{1},{\widetilde{X}}_{2},{\widetilde{X}}_{3}\right]=\left(2\pi /a\right)\mathbf{x}$, where *a* is a lattice constant, $s=0.0578$ is the shift coefficient that describes the translation of the yellow SG, and ${\mathbf{a}}_{s}={\mathbf{a}}_{1}+{\mathbf{a}}_{2}+2{\mathbf{a}}_{3}$ is the translation direction. For the inequalities marked in Fig. 3a, the level-set values ${f}_{{D}_{2}}=1.15$ and ${f}_{O}=1.1$ are used that determine the volume fraction of each SG. The refractive indices of the two SGs are commonly 4.0, and the outside region of them is filled with air whose refractive index is 1.0.

The perturbation strength $p$ plays an important role in our work. If $p$ becomes zero, the space group of this DG is $Ia\overline{3 }d$ (no. 230), and this does not exhibit Weyl points^18^. A DG with appropriate nonzero-valued $p$ exhibits four Weyl points in the momentum space between the fourth and fifth bands^17^. Due to the inversion symmetry of the momentum space, the positions of these four Weyl points are inversion symmetric to $\Gamma$-point. When $p={p}_{0}=0.195$ is used, we can get the four Weyl points, as shown in Fig. 3b,c. They are on a single $\left(001\right)$-plane passing $\mathrm{\Gamma N}=\left(-{\mathbf{b}}_{1}+{\mathbf{b}}_{2}\right)/2$ and $\mathrm{\Gamma H}=\left({\mathbf{b}}_{1}+{\mathbf{b}}_{2}-{\mathbf{b}}_{3}\right)/2$ where the reciprocal primitive vectors ${\mathbf{b}}_{i}$ are defined by ${\left[{\mathbf{b}}_{1}{\mathbf{b}}_{2}{\mathbf{b}}_{3}\right]}^{{\text{T}}}={2\pi \left[{\mathbf{a}}_{1}{\mathbf{a}}_{2}{\mathbf{a}}_{3}\right]}^{-1}$. These Weyl points are also denoted as ${{\text{N}}}_{0}$ and ${{\text{H}}}_{0}$ in Fig. 3d. The Chern numbers of the Weyl points ${{\text{N}}}_{0}$ and ${{\text{H}}}_{0}$ are $-1$ and $+1$, respectively (see Sect. 3, Supplementary Information).

### DG photonic array for pseudomagnetic field

First, we assume 48 DGs along the ${\mathbf{a}}_{\perp }$-direction between two parallel perfect electric conductor (PEC) boundaries, where ${\mathbf{a}}_{\perp }={\mathbf{a}}_{2}+0.5\left({\mathbf{a}}_{1}+{\mathbf{a}}_{3}\right)$ is normal to the boundaries (see Fig. 4a). The array is periodic along ${\mathbf{a}}_{=}$- and ${\mathbf{a}}_{\parallel }$-directions, where ${\mathbf{a}}_{=}=-{\mathbf{a}}_{1}+{\mathbf{a}}_{3}$ and ${\mathbf{a}}_{\parallel }={\mathbf{a}}_{1}+{\mathbf{a}}_{3}$. The boundaries are parallel to ${\mathbf{a}}_{1}$ and ${\mathbf{a}}_{3}$, while the $\left(001\right)$-plane shown in Fig. 4b is parallel to ${\mathbf{a}}_{1}$ and ${\mathbf{a}}_{2}$ (see Fig. S6 in Sect. 4, Supplementary Information). Thus, the boundaries and $\left(001\right)$-plane are neither parallel nor perpendicular. The Weyl points ${{\text{N}}}_{0}$ and ${{\text{H}}}_{0}$ on the $\left(001\right)$-plane is partially conserved by projecting it onto the boundary, as shown in Fig. 4b. Then, the perturbation strength is given by the position-dependent form, i.e., $p=p\left(\mathbf{x}\right)$. It linearly varies with the distance from the boundary at $n=0$ along the ${\mathbf{a}}_{\perp }$, i.e., ${\nabla }_{\mathbf{x}}p\propto {\mathbf{a}}_{\perp }$, and it equals ${p}_{0}$ at the midplane between the boundaries, as schematically illustrated in the inset of Fig. 4a. The DGs in the primitive cells in Fig. 4a exhibit different shapes. Especially, the yellow parts show a stronger defect-like shape with larger $p$.

The reasons that we utilized the PEC boundary are (1) the PEC boundary corresponds to a metal cap, and (2) implementation of the PEC boundary requires less computations than using a band gap material outside the boundaries.

To calculate Figs. 5 and 6, we use ${p}_{s}=\pm {p}_{s,0}$ where ${p}_{s,0}=2.9463\times {10}^{-4}{a}^{-1}$. The details not mentioned here are the same as the explanations in Sect. 1, Supplementary Information.

### Pseudomagnetic field by DG photonic array

Let us assume that the effective Hamiltonian around a Weyl point is expressed as ${H}_{eff}={\sum }_{i,j}^{3}{v}_{ij}\left({k}_{i}-{k}_{w}\right){\sigma }_{j}$ where ${\mathbf{k}}_{{\text{w}}}$ is the Weyl points locations (${\mathbf{k}}_{{\text{w}}}^{{\text{H}}}$ or ${\mathbf{k}}_{{\text{w}}}^{{\text{N}}}$), ${v}_{ij}$ is the velocity tensor, and ${{\varvec{\upsigma}}}_{j}$ are the Pauli matrices^13,15^. ${\mathbf{k}}_{{\text{w}}}$ can be decomposed into ${\mathbf{k}}_{{\text{w}}}={\mathbf{k}}_{{\text{w}},0}+{\widehat{\mathbf{k}}}_{{\text{s}}}\updelta k={\mathbf{k}}_{{\text{w}},0}+\mathbf{A}$ where the superscripts ${\text{N}}$ or ${\text{H}}$ of all terms are omitted. Only the last term $\mathbf{A}$ relies on the real space coordinate-dependent $p$, i.e., $\mathbf{A}=\mathbf{A}\left(p\left(\mathbf{x}\right)\right)$. From the information in Fig. 4a,b, the resulting pseudomagnetic field is, therefore, written as

6 $$\mathbf{B}={\nabla }_{\mathbf{x}}\times \mathbf{A}={p}_{s}\left({B}_{=}{\widehat{\mathbf{a}}}_{=}+{B}_{\parallel }{\widehat{\mathbf{a}}}_{\parallel }\right)$$

where ${\widehat{\mathbf{a}}}_{=}$ and ${\widehat{\mathbf{a}}}_{\parallel }$ are the unit vectors of ${\mathbf{a}}_{=}$ and ${\mathbf{a}}_{\parallel }$, respectively, placed on the boundary (see Fig. 4a). The components ${B}_{=}$ and ${B}_{\parallel }$ are determined by the trajectories in Fig. 3d,e. Meanwhile, the proportional constant ${p}_{s}$ is the gradient of geometrical non-uniformity and is defined by the ratio of the $p$ change to the distance between these two boundaries (see the inset in Fig. 4a). (Detailed derivations and explanations of this result are given in Sect. 4, Supplementary Information.) Because the length between the two boundaries can be written in terms of the lattice constant $a$, the proportional constant ${p}_{s}$ can be expressed in terms of ${a}^{-1}$.

The pseudomagnetic field $\mathbf{B}$ in Eq. (6) has the linear combination form of ${\widehat{\mathbf{a}}}_{=}$ and ${\widehat{\mathbf{a}}}_{\parallel }$, parallel to the boundaries, so the field is parallel to the boundaries, as marked in Fig. 4c,d. In other words, the common perpendicular direction of ${\widehat{\mathbf{a}}}_{=}$ and ${\widehat{\mathbf{a}}}_{\parallel }$ coincides with the surface normal to the boundaries. As a result, we can quantitate the overall Hall effect driven by the pseudomagnetic field $\mathbf{B}$ and the resultant wave localization around boundaries.

The photonic behavior when a pseudomagnetic field with a nonlinear perturbation is described in Sect. 5, Supplementary Information.

### Evasion of the photonic wave

**Figure 9:**
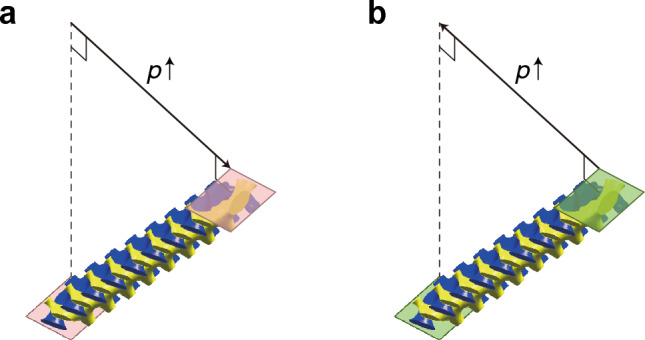
DG primitive cells array that was input to get Fig. 7a,b in the main text. The array consists of 8 primitive cells with spatially linearly decreasing or increasing $p$ along ${\widehat{\mathbf{a}}}_{\perp }$-direction.

Like the other photonic band structure calculations in this study, Fig. 7a,b is calculated using the array that consists of DG primitive cells, as illustrated in Fig. 9. The perturbation strength slope is counted by ${p}_{s,0}=7.0711\times {10}^{-3}{a}^{-1}$, e.g., ${p}_{s}=3{p}_{s,0}$. Figure 7d–g are calculated using the array that consists of big cells whose lattice vectors are $2{\mathbf{a}}_{\perp }$, ${\mathbf{a}}_{=}$, and ${\mathbf{a}}_{\parallel }$, where ${\mathbf{a}}_{\perp }=\left(a/2\right)\left[\mathrm{1,0},1\right]$, ${\mathbf{a}}_{=}=-{\mathbf{a}}_{1}+{\mathbf{a}}_{3}=a\left[\mathrm{1,0},-1\right]$, and ${\mathbf{a}}_{\parallel }={\mathbf{a}}_{1}+{\mathbf{a}}_{3}=a\left[\mathrm{0,1},0\right]$, respectively. We regard that the one big cell coincides with two DG primitive cells because the projections of ${\mathbf{a}}_{2}$ and ${\mathbf{a}}_{3}$ onto the plane of ${\widehat{\mathbf{a}}}_{\perp }-{\widehat{\mathbf{a}}}_{=}$ are half of $2{\mathbf{a}}_{\perp }$ and ${\mathbf{a}}_{=}$, respectively. Thus, the array marked with $m=32$ and $n=8$ in Fig. 7c consists of 16 and 4 big cells along ${\widehat{\mathbf{a}}}_{=}$ and ${\widehat{\mathbf{a}}}_{\perp }$-directions, respectively. Along with ${\mathbf{a}}_{\parallel }$, only one cell layer was used with periodic boundary conditions. To get Fig. 7d–g, the ‘Frequency Domain’ solver of COMSOL Multiphysics® was used with an input frequency of $0.49\left(2\pi c/\omega a\right)$, marked in Fig. 7a,b in the main text.

### Simulation details

All photonic structure simulations were performed using COMSOL Multiphysics®. To input periodicity, the Floquet periodic boundary condition was imposed on all periodic boundaries. All band structures were obtained using the ‘Eigenfrequency’ solver. In each band structure, we plot only nine bands above and below the zeroth Landau level so that only 19 bands are displayed in a band structure.

### Supplementary Information

Supplementary Information.

## Data Availability

The datasets generated during this study are available from the corresponding author on reasonable request.

## Code availability

The custom codes used in this study are available from the corresponding author on reasonable request.
