# Supplementary material for: Surface potential-adjusted surface states in 3D topological photonic crystals
Source: Sci Rep. 2024 Mar 26;14:7173. doi: 10.1038/s41598-024-56894-6 (PMC11344842; doi:10.1038/s41598-024-56894-6)
Supplement: Supplementary file 1 — Supplementary Information. [file 41598_2024_56894_MOESM1_ESM.docx]

Supplementary Information for

**“Surface potential-adjusted surface states in 3D topological photonic crystals”**

Haedong Park^1,2^, Sang Soon Oh^2^*, and Seungwoo Lee^1,3,4,5*^

^1^KU-KIST Graduate School of Converging Science and Technology, Korea University, Seoul 02841, Republic of Korea

^2^School of Physics and Astronomy, Cardiff University, Cardiff CF24 3AA, United Kingdom

^3^Department of Biomicrosystem Technology, Korea University, Seoul 02841, Republic of Korea

^4^Department of Integrative Energy Engineering and KU Photonics Center, Korea University, Seoul, 02841 Republic of Korea

^5^Center for Opto-Electronic Materials and Devices, Post-Silicon Semiconductor Institute, Korea Institute of Science and Technology (KIST), Seoul 02792, Republic of Korea

*Email: ohs2@cardiff.ac.uk, seungwoo@korea.ac.kr

**Contents**

| 1. Photonic Landau levels and photonic wave localization | - - - - - - - - - - - - - - - - - - | 3 |
| --- | --- | --- |

| 2. Surface states by Weyl equation | - - - - - - - - - - - - - - - - - - - - - - - - - - - - - - - - | 6 |
| --- | --- | --- |

| 3. Berry phase of Weyl points at equifrequency | - - - - - - - - - - - - - - - - - - - - - - - - - | 11 |
| --- | --- | --- |

| 4. Derivation of pseudomagnetic field in DG array | - - - - - - - - - - - - - - - - - - - - - - | 12 |
| --- | --- | --- |

| 5. Pseudomagnetic field with a nonlinear perturbation | - - - - - - - - - - - - - - - - - - - - - - | 16 |
| --- | --- | --- |

| 6. Remarks on experimental realization | - - - - - - - - - - - - - - - - - - - - - - - - - - - - - - | 17 |
| --- | --- | --- |

| 7. Additional data about the evasion behavior of photonic waves | - - - - - - - - - - - - | 19 |
| --- | --- | --- |

| 8. Effective surface potential regarding a double gyroid array | - - - - - - - - - - - - - - - | 20 |
| --- | --- | --- |

**1. Photonic Landau levels and photonic wave localization**

Applying the design in Fig. 4a in the main text to the array of 48 primitive cells generates Landau levels and Landau plateau near $\omega_{0}$ (Fig. S1). The perturbation strength $p$ linearly increases from $n=0$ to $n=48$. We display all these Landau spectrums along with the $\Gamma N^{'}$-(Fig. S1a-c) and $\Gamma H^{'}$-directions (Fig. S1d-f) ($\Gamma N^{'}$ and $\Gamma H^{'}$ are marked in the inset in Fig. S1d). The width of the Landau plateau is a scale of the magnetic lengths, $l_{N}^{-1}\sim\sqrt{B^{N}}$ and $l_{H}^{-1}\sim\sqrt{B^{H}}$, and marked in Fig. S1a and d, respectively. We show the results only on positive $\Gamma N^{'}$- and $\Gamma H^{'}$-directions because all eigenfrequencies and eigenstates are even at the $\Gamma$-point, due to time-reversal symmetry. We investigate the eigenstates along the zeroth Landau level (red curves in Fig. S1a and d) to see surface states localized on the boundary. The process of collecting eigenstates is as follows: first, for a given $\mathbf{k}$, we gather the average normal values of magnetic field eigenstates in each unit cell. These values are normalized with the maximum among these. We then collect all these sets for the $\mathbf{k}$ along the $\Gamma N^{'}$- and $\Gamma H^{'}$-axes, as shown in Fig. S1b and e, respectively. Localization aspects vary significantly by the $N_{0}$ or $H_{0}$. For $\left| \mathbf{k} \right|<\left| \Gamma N_{0} \right|$ and $\left| \mathbf{k} \right|<\left| \Gamma H_{0} \right|$, the eigenstates are localized on the lower boundary (which corresponds to the leftmost boundary in Fig. 4a in the main text). The confinement length on the lower boundary is a scale of the magnetic length. Around $N_{0}$ and $H_{0}$, eigenstates vary dramatically with increasing $\mathbf{k}$. For $\left| \mathbf{k} \right|>\left| \Gamma N_{0} \right|$ and $\left| \mathbf{k} \right|>\left| \Gamma H_{0} \right|$, we observe the eigenstates localized on the upper boundary (which corresponds to the rightmost boundary in Fig. 4a in the main text). These results show that $N_{0}$ and $H_{0}$, the Weyl points with $p=p_{0}$, are the phase transition points. We carried out the same computations with several values of $p_{s}$. The zeroth Landau level flattens over a larger momentum interval as the pseudomagnetic field increases (Fig. S1c and f).


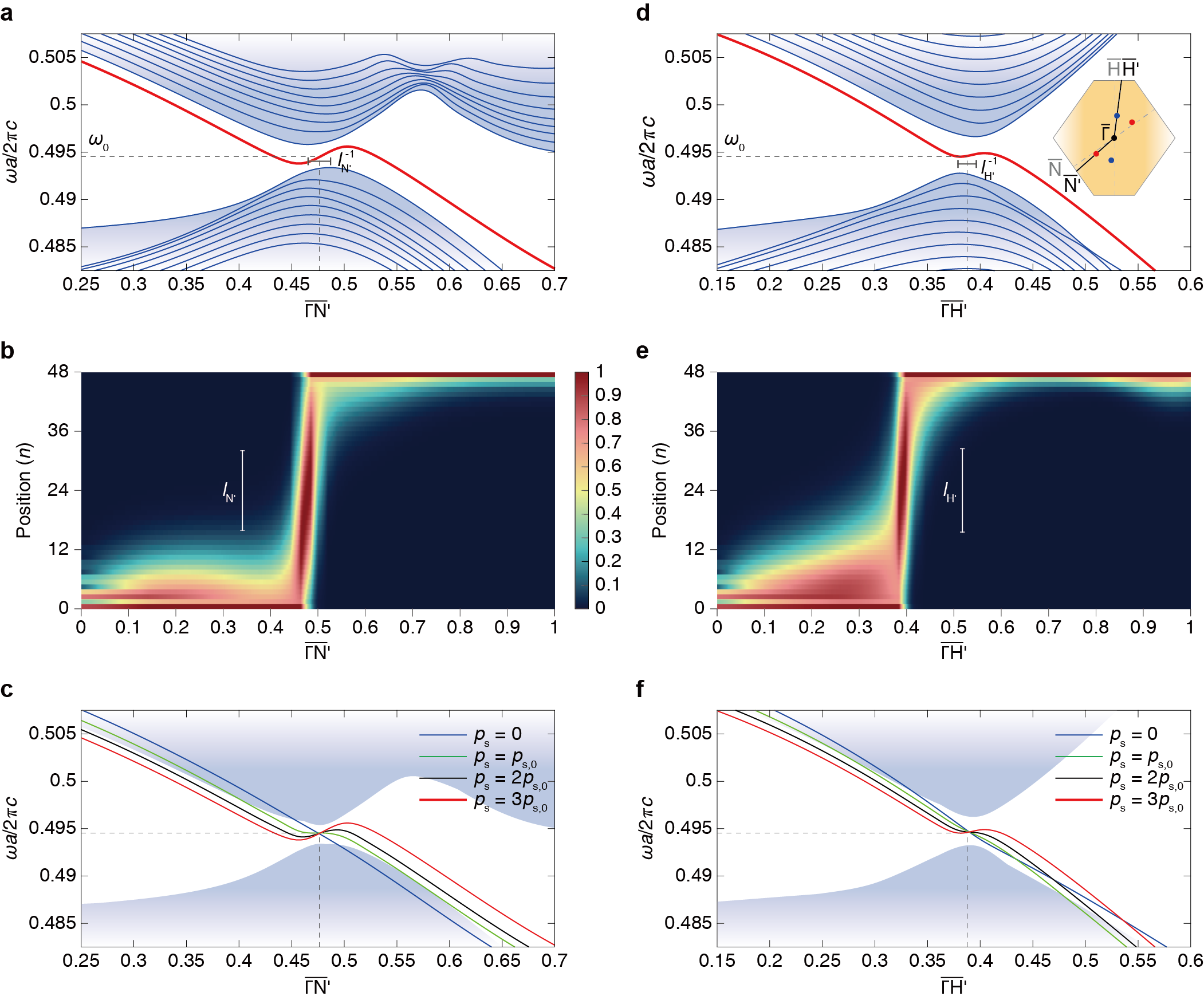


**Fig. S1. Photonic Landau levels and eigenstates in a pseudomagnetic field by a spatial gradient of** $p$**. a** and **d**, Photonic band structures along $\Gamma N^{'}$- (**a**) and $\Gamma H^{'}$-directions (**d**), which exhibit Landau plateaus at around $N_{0}$ and $H_{0}$, respectively. Here, $p_{s}=3p_{s,0}$ is used where $p_{s,0}=2.9463\times{10}^{-4}a^{-1}$. **b** and **e**, Normalized eigenstates along the zeroth Landau levels (red curves in **a** and **d**, respectively) to see surface states localized on the boundary. **c** and **f**, Zeroth Landau levels according to several magnitudes of pseudomagnetic fields. The $\Gamma N^{'}$ and $\Gamma H^{'}$-directions are marked in the inset of **d**. All the plots are simulation results.

Although we plot the zeroth Landau levels for several values of $p_{s}$ including $p_{s}=0$, we need to think about whether the pseudomagnetic field is truly essential for generating these surface states. One difficulty of observing the surface states with $p_{s}=0$ is that the surface band is attached to or buried into bulk bands, as shown in Fig. S2. To observe the effect of a surface potential on the surface band, the surface band needs to be detached from the bulk bands. In other words, thanks to the pseudomagnetic field, we can expose the effects of a surface potential clearly.


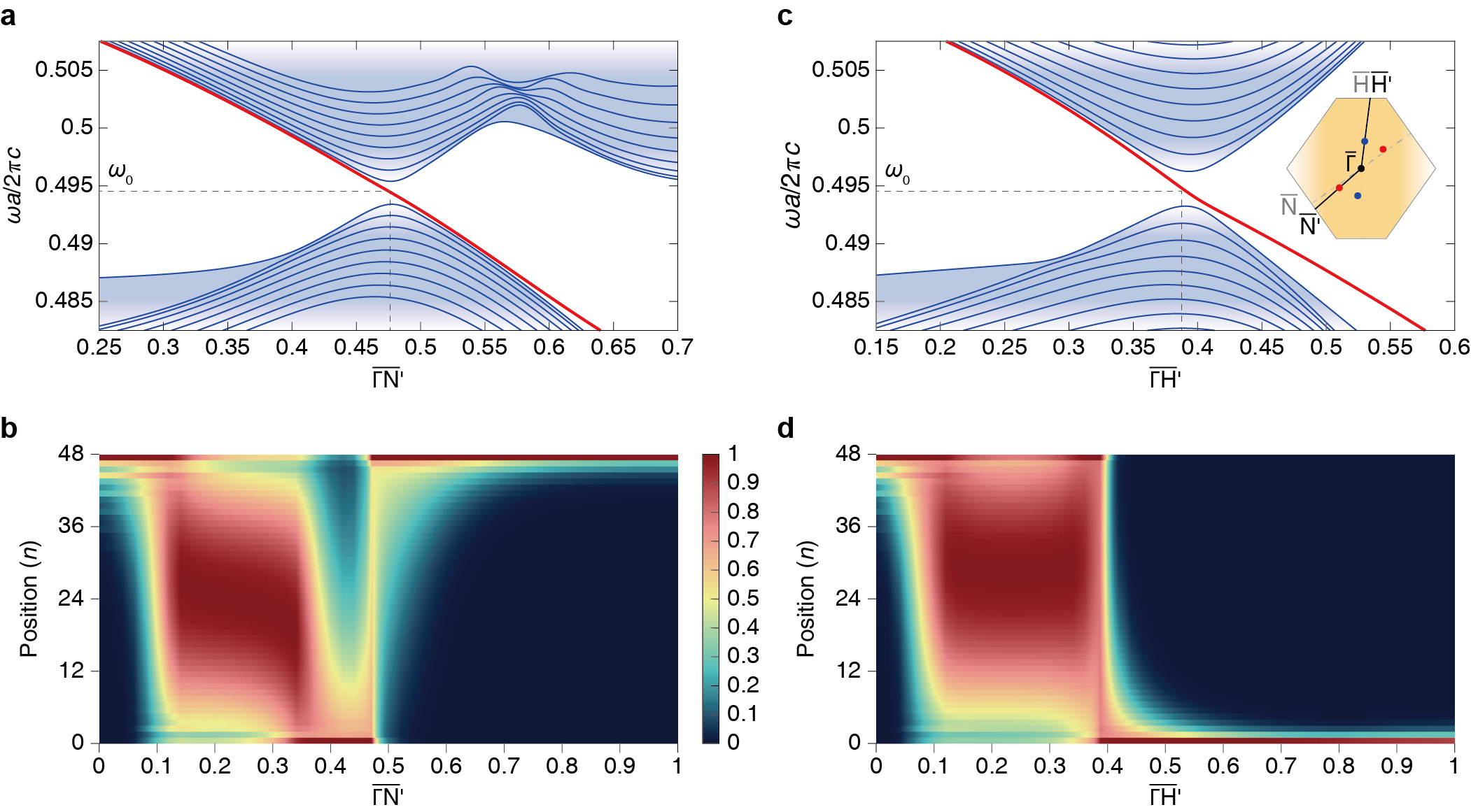


**Fig. S2. Photonic Landau levels and eigenstates in a pseudomagnetic field by a spatial gradient of** $\boldsymbol{p}$ **when** $\boldsymbol{p}_{\boldsymbol{s}}\boldsymbol{=0}$**. a-b**, Photonic band structures along $\Gamma N^{'}$-direction and normalized eigenstates along the zeroth Landau level (red curve in **a**), respectively. **c-d**, Photonic band structures along $\Gamma H^{'}$-direction and normalized eigenstates along the zeroth Landau level (red curve in **c**), respectively. The $\Gamma N^{'}$ and $\Gamma H^{'}$-directions are marked in the inset of **c**. All the plots are simulation results.

**2. Surface states by Weyl equation**

Based on equation (4) in the main text, we give detailed derivation of the formula exhibiting surface states. For an orthogonal coordinate system by $x_{1}$, $x_{2}$, and $x_{3}$, we consider a system periodic along with $x_{1}$- and $x_{2}$-directions, and finite along $x_{3}$-direction, as mentioned in the main text. Thus, between the surface boundaries parallel to $x_{1}$- and $x_{2}$-directions, there are $N$ cells along $x_{3}$-direction. One boundary’s coordinate is considered as $\left[ x_{1},x_{2},0 \right]$, and the other is $\left[ x_{1},x_{2},Na_{3} \right]$, where $a_{3}=\left| \mathbf{a}_{3} \right|$ is the $x_{3}$-directional lattice constant. A point $\mathbf{x}\mathbf{=}\left[ x_{1},x_{2},x_{3} \right]$ in $n$th grid from the first boundary is discretized as $\left[ x_{1},x_{2},na_{3} \right]$. The traveling wave solution $\psi\left( \mathbf{x},t \right)=\psi_{0}e^{i\left( \mathbf{k}\cdot\mathbf{x}-\omega t \right)}$ can be written as

|  | $\psi\left( \mathbf{x},t \right)=u\left( x_{3} \right)e^{i\left( k_{1}x_{1}+k_{2}x_{2}-\omega t \right)}=u_{n}e^{i\left( k_{1}x_{1}+k_{2}x_{2}-\omega t \right)} ,$ | (S1) |
| --- | --- | --- |

where $u_{n}=u\left( x_{3}=na_{3} \right)$. Substituting this solution in the equation (4) in the main text gives

|  | $\left\{ \left( k_{1}-k_{w,1} \right)\sigma_{1}+\left( k_{2}-k_{w,2} \right)\sigma_{2}-k_{w,3}\sigma_{3} \right\}u_{n}-i\sigma_{3}\frac{\partial u_{n}}{\partial x_{3}}+V_{s}\sigma_{3}u_{n}=\omega u_{n} ,$ | (S2) |
| --- | --- | --- |

where $\mathbf{k}_{w}=\left[ k_{w,1},k_{w,2},k_{w,3} \right]$ is the Weyl point’s location, which can be decomposed as $\mathbf{k}_{w}=\mathbf{k}_{w,0}+\mathbf{A}_{w}^{n}$. Thus, $\mathbf{k}_{w}$ depends on the grid index $n$. The scalar coefficient of the surface potential $V_{s}$ also varies with $n$. For $1<n<N$, ${\partial u_{n}}/{\partial x_{3}}$ is numerically converted as

|  | $\frac{\partial u_{n}}{\partial x_{3}}=\frac{u_{n+1}-u_{n-1}}{2a_{3}} .$ | (S3) |
| --- | --- | --- |

Thus, equation (S2) becomes

|  | $Du_{n-1}+C_{n}u_{n}-Du_{n+1}+V_{s}\sigma_{3}u_{n}=\omega u_{n} ,$ | (S4) |
| --- | --- | --- |

where

|  | $C_{n}=\left( k_{1}-k_{w,1} \right)\sigma_{1}+\left( k_{2}-k_{w,2} \right)\sigma_{2}-k_{w,3}\sigma_{3}$ | (S5) |
| --- | --- | --- |

and

|  | $D=\frac{i\sigma_{3}}{2a_{3}} .$ | (S6) |
| --- | --- | --- |

Here, $C$ has a subscript $n$ due to $\mathbf{k}_{w}$ varies with $n$. Around the boundaries i.e., $n=1$ and $n=N$, we use following numerical differentials:

|  | $\frac{\partial u_{1}}{\partial x_{3}}=\frac{-3u_{1}+4u_{2}-u_{3}}{2a_{3}}$ | (S7) |
| --- | --- | --- |

and

|  | $\frac{\partial u_{N}}{\partial x_{3}}=\frac{u_{N-2}-4u_{N-1}+3u_{N}}{2a_{3}} .$ | (S8) |
| --- | --- | --- |

By substituting equations (S7) and (S8) into (S2), we have

|  | $\left( C_{1}+3D \right)u_{1}-4Du_{2}+Du_{3}+V_{s}\sigma_{3}u_{1}=\omega u_{1}$ | (S9) |
| --- | --- | --- |

and

|  | $-Du_{N-2}+4Du_{N-1}+\left( C_{N}-3D \right)u_{N}+V_{s}\sigma_{3}u_{N}=\omega u_{N} ,$ | (S10) |
| --- | --- | --- |

respectively. Equations (S4), (S9), and (S10) are summarized as follows:

| $\left[ \begin{matrix} C_{1}+3D & -4D & D & 0 & \cdots& 0 & 0 & 0 & 0 \\ D & C_{2} & -D & 0 & \cdots& 0 & 0 & 0 & 0 \\ 0 & D & C_{3} & -D & \cdots& 0 & 0 & 0 & 0 \\ \vdots& \vdots& \vdots& \vdots& \ddots& \vdots& \vdots& \vdots& \vdots\\ 0 & 0 & 0 & 0 & \cdots& D & C_{N-2} & -D & 0 \\ 0 & 0 & 0 & 0 & \cdots& 0 & D & C_{N-1} & -D \\ 0 & 0 & 0 & 0 & \cdots& 0 & -D & 4D & C_{N}-3D \end{matrix} \right]\left[ \begin{matrix} u_{1} \\ u_{2} \\ u_{3} \\ \vdots\\ u_{N-2} \\ u_{N-1} \\ u_{N} \end{matrix} \right]$  $+\left[ \begin{matrix} V_{s}\sigma_{3} & 0 & 0 & \cdots& 0 & 0 & 0 \\ 0 & V_{s}\sigma_{3} & 0 & \cdots& 0 & 0 & 0 \\ 0 & D & V_{s}\sigma_{3} & \cdots& 0 & 0 & 0 \\ \vdots& \vdots& \vdots& \ddots& \vdots& \vdots& \vdots\\ 0 & 0 & 0 & \cdots& V_{s}\sigma_{3} & 0 & 0 \\ 0 & 0 & 0 & \cdots& 0 & V_{s}\sigma_{3} & 0 \\ 0 & 0 & 0 & \cdots& 0 & 0 & V_{s}\sigma_{3} \end{matrix} \right]\left[ \begin{matrix} u_{1} \\ u_{2} \\ u_{3} \\ \vdots\\ u_{N-2} \\ u_{N-1} \\ u_{N} \end{matrix} \right]=\omega\left[ \begin{matrix} u_{1} \\ u_{2} \\ u_{3} \\ \vdots\\ u_{N-2} \\ u_{N-1} \\ u_{N} \end{matrix} \right] .$ | | |
| --- | --- | --- |
|  |  | (S11) |

For a system with $N=48$, we simply use $\mathbf{a}_{i}=a{\hat{\mathbf{x}}}_{i}$ and $\left[ \mathbf{b}_{1}\mathbf{b}_{2}\mathbf{b}_{3} \right]^{T}={2\pi\left[ \mathbf{a}_{1}\mathbf{a}_{2}\mathbf{a}_{3} \right]}^{-1}$. We set the constant term of the Weyl point’s location $\mathbf{k}_{w,0}$ as $\mathbf{k}_{w,0}=0.194\mathbf{b}_{1}-0.22\mathbf{b}_{2}+0.1171\mathbf{b}_{3}$. The varying term $\mathbf{A}_{w}^{n}$ is written as $\mathbf{A}_{w}^{n}=p_{s}\left\{ \left( n-1 \right)a_{3}-d_{0} \right\}\mathbf{k}_{s}$, where $\mathbf{k}_{s}=\left[ 6.4867, \right. -5.5920, \left. 1.4216 \right]a^{-1}$ is the Weyl point’s shift direction with varying $n$, and $d_{0}=\left( N/2-1/2 \right)a_{3}$ is the distance between the midplane and the $n=1$ grid.

We first investigate the surface localization when the surface potential is not considered (i.e., $V_{s}$ is zero for all the grids). When $p_{s}=-p_{s,0}$ and $p_{s}=p_{s,0}$ were respectively used ($p_{s,0}=8.8388\times{10}^{-4}a^{-1}$), the zeroth Landau levels shown in Fig. S3a-b are identical. We collect the (normalized) eigenstates intensities along with the zeroth Landau levels to observe surface states localized on the boundaries. As shown in Fig. S3c-f, the eigenstate amplitudes are mutually symmetric.

On the contrary, when we consider the $V_{s}$, the mutual symmetric distributions of wave intensities for the two cases are broken. We assume that $V_{s}$ is non-zero only on the first/last one or two grids around the boundaries, as shown in Fig. 8 in the main text; $V_{s}\left( n=1 \right)=-0.1a^{-1}$, $V_{s}\left( n=47 \right)=0.05a^{-1}$, and $V_{s}\left( n=48 \right)=0.1a^{-1}$. Although several studies^1-5^ have used the Dirac delta function or a similar function as $V_{s}$, we impose finite values as we consider the finite and discretized system. The overall shape of $V_{s}=V_{s}\left( n \right)$ is similar to Fig. 1d in the main text. The resulting surface localization is biased towards a specific boundary, as shown in Fig. S4.


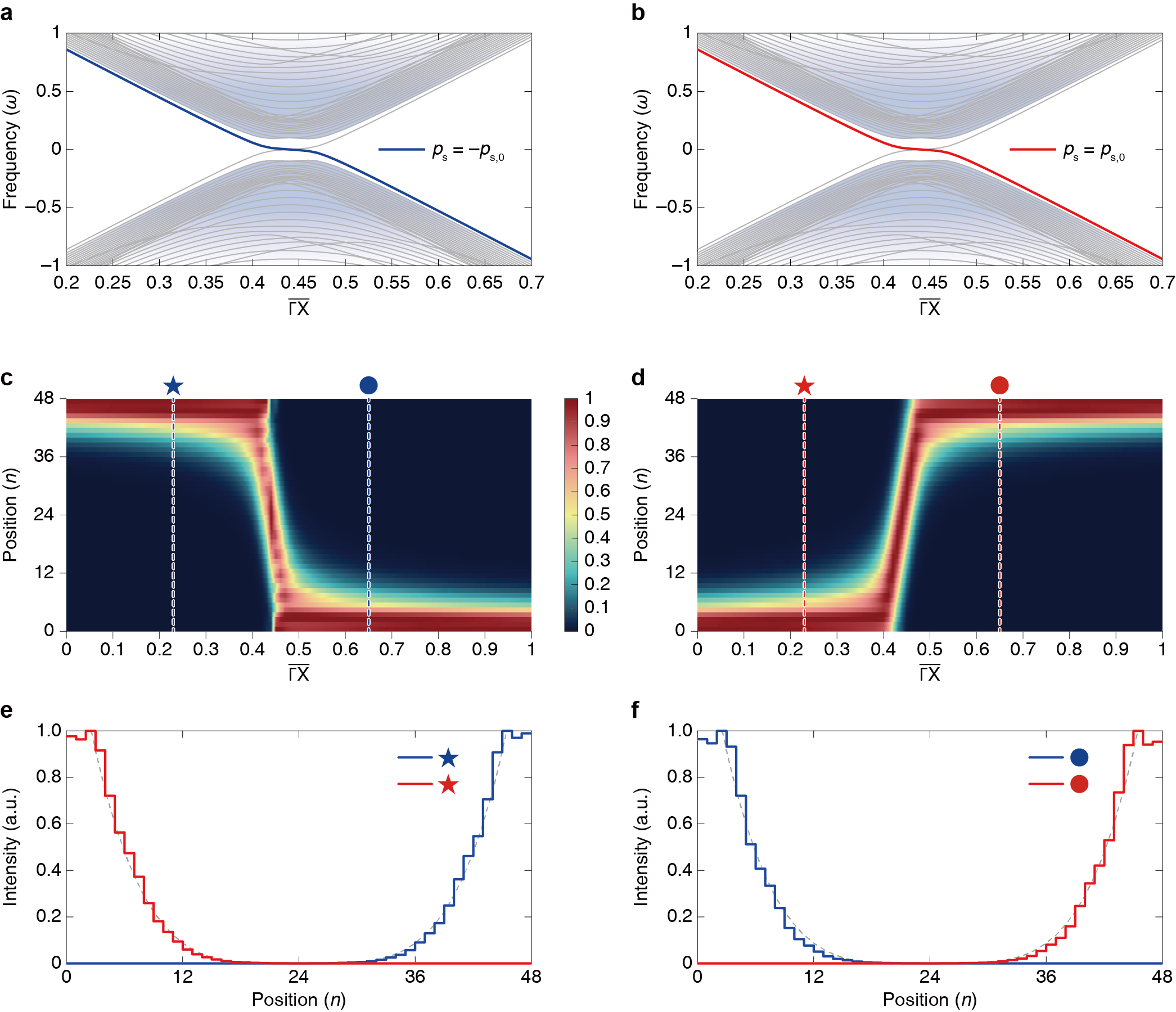


**Fig. S3. Symmetric localization of eigenstates by Weyl equation when pseudomagnetic field is applied. a**, **b**, Band structures and the zeroth Landau levels calculated by equation (S11). Each case corresponds to $p_{s}=-p_{s,0}$ and $p_{s}=p_{s,0}$, respectively, where $p_{s,0}=8.8388\times{10}^{-4}a^{-1}$. $\bar{\Gamma X}=0.441\mathbf{b}_{1}-0.5\mathbf{b}_{2}$ passes the projected Weyl point’s location. **c**, **d**, Normalized eigenstates along the zeroth Landau levels shown in **a** and **b** to see surface states localized on the boundaries. **e**, **f**, Comparisons of the localized wave intensities along the vertical lines marked in **c** and **d**. Data with the same symbols are overlapped in the same plot. Gray dotted lines are the symmetric curves with respect to $n=24$ for comparisons of the red and blue plots.


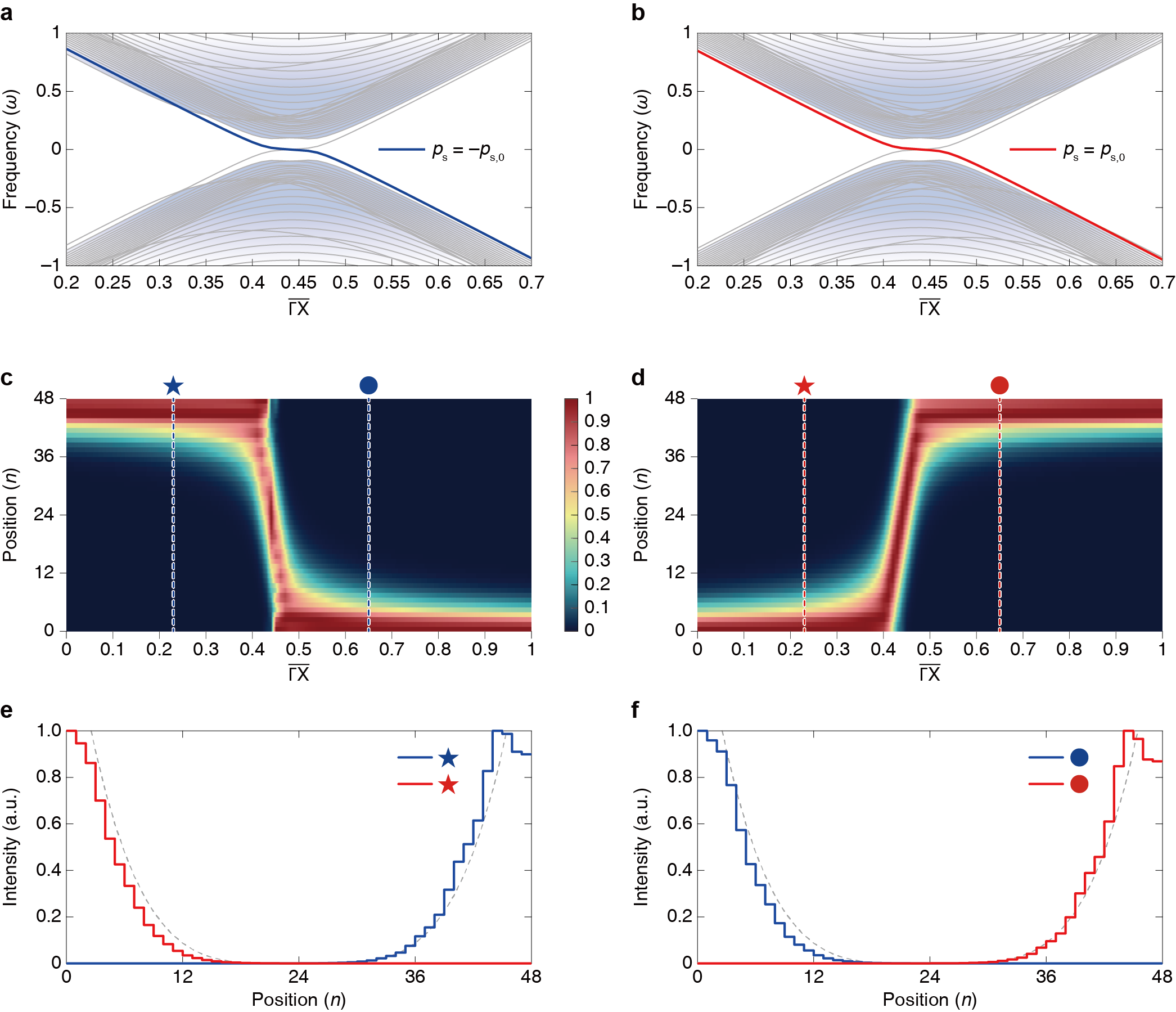


**Fig. S4. Asymmetric localization of eigenstates by Weyl equation when pseudomagnetic field and surface potential are applied.** The explanations for Fig. S3 can be used again for all the panels. The main point in here is that the wave intensities are biased towards the $n=0$ boundary due to the application of the surface potential.

**3. Berry phase of Weyl points at equifrequency**

We introduced a double gyroid (DG) photonic crystal in Fig. 3 in the main text. The DG exhibits four point-degeneracies marked as $N_{0}$ and $H_{0}$ in Fig. 3d in the main text. To see if these are Weyl points, we calculate the Berry phase using the Wilson Loop method^6-10^. The Berry phases of the lower and upper bands connected to point $N_{0}$ exhibit decreasing and increasing by $2\pi$, respectively (see Fig. S5a). Therefore, its Chern number is $-1$. Likewise, the lower and upper bands connected to point $H_{0}$ reveals increasing and decreasing by $2\pi$, respectively (see Fig. S5b). Therefore, its Chern number is +$1$. All these are nonzero Chern numbers. Thus, these points are Weyl points, and all surface states in this study are topologically nontrivial by the Weyl points.


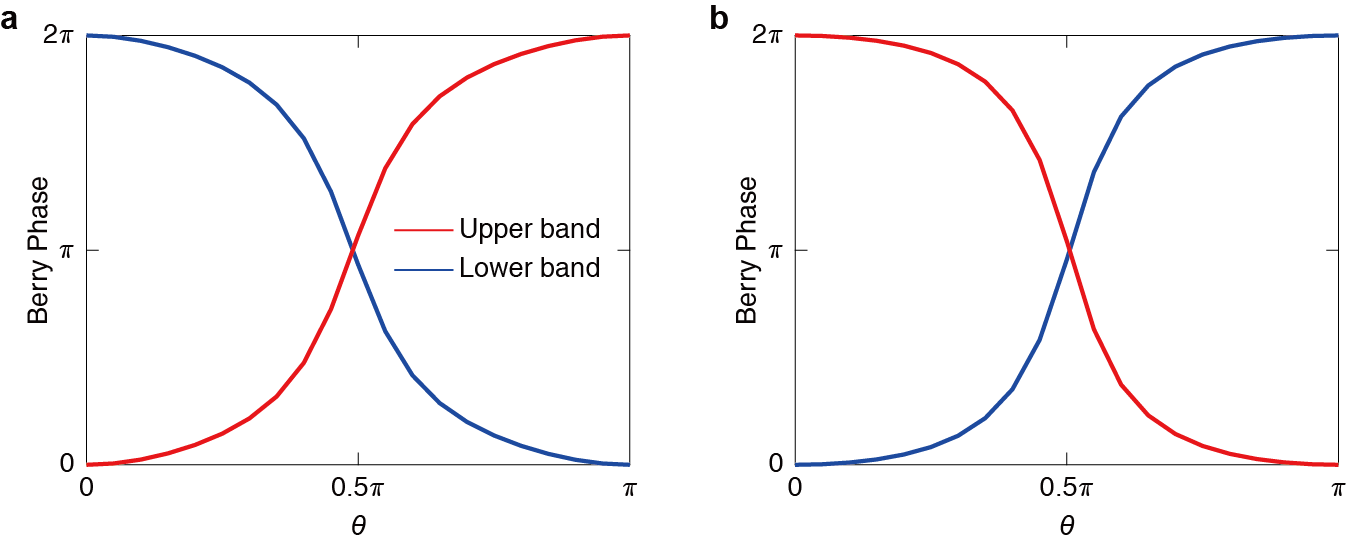


**Fig. S5. Berry phase around the equifrequency Weyl points** $N_{0}$ **(a) and** $H_{0}$ **(b) shown in Fig. 1e in the main text.** The Chern numbers of $N_{0}$ and $H_{0}$ are $-1$ and $+1$, respectively.

**4. Derivation of pseudomagnetic field in DG array**

Here, we use $\mathbf{a}_{1}=a/2\left[ -1,1,1 \right]$, $\mathbf{a}_{2}=a/2\left[ 1,-1,1 \right]$, and $\mathbf{a}_{3}=a/2\left[ 1,1,-1 \right]$. The normal vector of the perfect electric conductor (PEC) boundary at $n=0$ shown in Fig. 4a in the main text is $\mathbf{a}_{\perp}=\mathbf{a}_{2}+0.5\left( \mathbf{a}_{1}+\mathbf{a}_{3} \right)$. We assume that this plane passes the origin. Therefore, the distance between a point $\mathbf{x}\mathbf{=}\left[ x_{1}\boldsymbol{,}x_{2}\boldsymbol{,}x_{3} \right]$ and this plane is $d\left( \mathbf{x} \right)={\hat{\mathbf{a}}}_{\perp}\cdot\left( \mathbf{x}-\boldsymbol{0} \right)$ or $d\left( \mathbf{x} \right)=\left( x_{1}+x_{3} \right)/\sqrt{2}$. The perturbation strength $p$, plotted in the inset of Fig. 2c in the main text, is given by

|  | $p\left( \mathbf{x} \right)=p_{s}\left\{ d\left( \mathbf{x} \right)-d_{0} \right\}+p_{0}$ | (S12) |
| --- | --- | --- |

where $p_{s}$ is a proportional constant, and $d_{0}$ is a distance between the planes at $n=0$ and $n=N/2$, marked in the inset of Fig. 4a in the main text. The constant $p_{0}$ is a central value of $p\left( \mathbf{x} \right)$ (see the inset of Fig. 4a in the main text) or a perturbation strength used for the default photonic crystal to generate Fig. 3a-c in the main text. If the number of unit cells between these boundaries is $N$, and the perturbation strengths at these boundaries has been determined, the proportional constant $p_{s}$ is calculated as follows:

|  | $p_{s}=\frac{p\left( n\mathbf{a}_{\perp} \right)-p\left( \mathbf{0} \right)}{d\left( n\mathbf{a}_{\perp} \right)-d\left( \mathbf{0} \right)}$ | (S13) |
| --- | --- | --- |

In other words, this quantity reflects the design of the DG array, such as the number of unit cells and the gradient of the perturbation strength.

As shown in Fig. 3d in the main text, Weyl points are confined to $\left( 001 \right)$-plane, irrespective of $p$. The normal direction of this plane coincides with the normal direction of the plane spanned by $\mathbf{a}_{1}$ and $\mathbf{a}_{2}$ (see Fig. S6). If we transform the coordinate system by

|  | $\mathbf{x}^{'}=\left[ \begin{matrix} \cos\frac{\pi}{4} & -\sin\frac{\pi}{4} & 0 \\ 0 & 0 & 1 \\ -\sin\frac{\pi}{4} & -\cos\frac{\pi}{4} & 0 \end{matrix} \right]\mathbf{x}$ | (S14) |
| --- | --- | --- |

or

|  | $\mathbf{x}=\left[ \begin{matrix} \cos\left( -\frac{\pi}{4} \right) & 0 & \sin\left( -\frac{\pi}{4} \right) \\ \sin\left( -\frac{\pi}{4} \right) & 0 & -\cos\left( -\frac{\pi}{4} \right) \\ 0 & 1 & 0 \end{matrix} \right]\mathbf{x}^{'}$ | (S15) |
| --- | --- | --- |

the directions of $\Gamma N$ and $\Gamma H$ become equivalent to ${\hat{\mathbf{x}}}_{1}^{'}=\left[ 1,0,0 \right]$ and ${\hat{\mathbf{x}}}_{2}^{'}=\left[ 0,1,0 \right]$, respectively, so that we can easily write a Hamiltonian for this system. (The relation between the directions of $\Gamma N$, $\Gamma H$, and $\mathbf{a}_{i}$ are marked in Fig. S6.) The $p$ with respect to the new coordinate system is given by

|  | $p\left( \mathbf{x'} \right)=p_{s}\left\{ \frac{\frac{1}{\sqrt{2}}\left( x_{1}^{'}-x_{3}^{'} \right)+x_{2}^{'}}{\sqrt{2}}-d_{0} \right\}+p_{0}$ | (S16) |
| --- | --- | --- |

The effective Hamiltonian around a Weyl point is expressed as^9,11^

|  | $H_{eff}=\sum_{i,j}^{3} v_{ij}\left( k_{i}-k_{w} \right)\sigma_{j}$ | (S17) |
| --- | --- | --- |

where *v_ij_* is the anisotropic velocity tensor, **k** is the wave vector, and **σ***_j_* is the Pauli matrices. $\mathbf{k}_{w}$ is $\mathbf{k}_{w}^{H}$ or $\mathbf{k}_{w}^{N}$. Here, the superscripts N or H of all terms are omitted. If the Weyl points behave like Fig. 3d in the main text, $\mathbf{k}_{w}$ can be decomposed into a constant term, $\mathbf{k}_{w,0}$, and a varying term, ${\hat{\mathbf{k}}}_{s}\delta k=\mathbf{A}$, i.e., $\mathbf{k}_{w}=\mathbf{k}_{w,0}+{\hat{\mathbf{k}}}_{s}\delta k=\mathbf{k}_{w,0}+\mathbf{A}$. For the Weyl points marked in Fig. 3d in the main text, the vector potential and pseudomagnetic field for the new coordinate system are respectively given by

|  | $\mathbf{A}^{\mathbf{'}}=\left[ k_{s,1}\left\{ p\left( \mathbf{x'} \right)-p_{0} \right\},k_{s,2}\left\{ p\left( \mathbf{x'} \right)-p_{0} \right\},0 \right]=\frac{p_{s}}{\sqrt{2}}\left\{ \frac{1}{\sqrt{2}}\left( x_{1}^{'}-x_{3}^{'} \right)+x_{2}^{'} \right\}\left[ k_{s,1},k_{s,2},0 \right]+C$ | (S18) |
| --- | --- | --- |

and

|  | $\mathbf{B}^{\mathbf{'}}=\nabla_{\mathbf{x}^{'}}\times\mathbf{A}^{\mathbf{'}}=\frac{p_{s}}{2}\left[ k_{s,2},-k_{s,1},k_{s,2}-\sqrt{2}k_{s,1} \right]=\frac{p_{s}k_{s,1}}{\sqrt{2}}\frac{\left[ 1,-\sqrt{2},-1 \right]}{2}-\frac{p_{s}}{\sqrt{2}}\left( k_{s,2}-\frac{k_{s,1}}{\sqrt{2}} \right)\frac{\left[ -1,0,-1 \right]}{\sqrt{2}}$ | (S19) |
| --- | --- | --- |

where $C=-p_{s}d_{0}\left[ k_{s,1},k_{s,2},0 \right]$ is a constant term. Proportional constants $k_{s,1}$ and $k_{s,2}$ are obtained from the traces in Fig. 3d-e in the main text. Thus, unlike $p_{s}$ in equation (S13), the constants $k_{s,1}$ and $k_{s,2}$ do not reflect the design of the array, but they are about the nature of a three-dimensional DG Weyl photonic crystal itself. The pseudomagnetic field with respect to the original coordinate system is written as follows:

|  | $\mathbf{B}=p_{s}\left\{ \frac{k_{s,1}}{\sqrt{2}}\frac{\left[ 1,0,-1 \right]}{\sqrt{2}}-\frac{1}{\sqrt{2}}\left( k_{s,2}-\frac{k_{s,1}}{\sqrt{2}} \right)\left[ 0,1,0 \right] \right\}=p_{s}\left( B_{=}{\hat{\mathbf{a}}}_{=}\boldsymbol{+}B_{\parallel}{\hat{\mathbf{a}}}_{\parallel} \right)$ | (S20) |
| --- | --- | --- |

where $B_{=}$ and $B_{\parallel}$ are respectively

|  | $B_{=}=\frac{k_{s,1}}{\sqrt{2}}$ | (S21) |
| --- | --- | --- |
|  | $B_{\parallel}=-\frac{1}{\sqrt{2}}\left( k_{s,2}-\frac{k_{s,1}}{\sqrt{2}} \right)$ | (S22) |

and ${\hat{\mathbf{a}}}_{=}\boldsymbol{=}\left[ 1,0,-1 \right]/\sqrt{2}$ and ${\hat{\mathbf{a}}}_{\parallel}\boldsymbol{=}\left[ 0,1,0 \right]$ are the unit vectors of $\mathbf{a}_{=}=-\mathbf{a}_{1}+\mathbf{a}_{3}$ and $\mathbf{a}_{\parallel}=\mathbf{a}_{1}+\mathbf{a}_{3}$, respectively, placed on the boundary (see Fig. 4a in the main text). The number of cells $N$ and the difference of $p\left( \mathbf{x} \right)$ at both boundaries are reflected only in $p_{s}$, as mentioned in equation (S13). Therefore, the direction of the pseudomagnetic field $\mathbf{B}$ is always fixed, regardless of the system scale and the gradient of $p\left( \mathbf{x} \right)$.

The values of each variable in the above derivations are summarized in **Table S1**. All these values are about the pseudomagnetic fields derived by Weyl points around $N_{0}$ and $H_{0}$ for $N=48$. Both pseudomagnetic fields at the antipodes of $N_{0}$ and $H_{0}$ are $-\mathbf{B}^{N}$ and $-\mathbf{B}^{H}$, respectively, due to the signs of $\mathbf{k}_{s}^{N}$ and $\mathbf{k}_{s}^{H}$ are flipped.

**Table S1. Information of pseudomagnetic fields derived by Weyl points around** $N_{0}$ **and** $H_{0}$ **for 48 cells.** All these are calculated by $p_{s}=8.8388\times{10}^{-4}a^{-1}$. The magnetic length is given by $l^{-1}\sim\sqrt{B}$.

| $\mathbf{k}_{s}^{N}=\left[ \begin{matrix} k_{s,1}^{N} & k_{s,2}^{N} \end{matrix} \right]=\left[ \begin{matrix} 7.9083 & -3.5815 \end{matrix} \right]a^{-1}$ (in $\mathbf{x}^{'}$-coordinate)  $\mathbf{B}^{N}=p_{s}B_{=}^{N}{\hat{\mathbf{a}}}_{=}\boldsymbol{+}p_{s}B_{\parallel}^{N}{\hat{\mathbf{a}}}_{\parallel}$  $=4.9427\times{10}^{-3}a^{-2}{\hat{\mathbf{a}}}_{=}\boldsymbol{+}5.7334\times{10}^{-3}a^{-2}{\hat{\mathbf{a}}}_{\parallel}$  $=\left[ \begin{matrix} 3.495 & 5.7334 & -3.495 \end{matrix} \right]\times{10}^{-3}a^{-2}$  $=\left[ \begin{matrix} 3.9541 & 6.4866 & -3.9541 \end{matrix} \right]p_{s}a^{-1}$ (in $\mathbf{x}$-coordinate)  $B^{N}=7.5698\times{10}^{-3}a^{-2}=8.5643p_{s}a^{-1}$  $l_{N}=11.4936a$  $l_{N}^{-1}=0.087005a^{-1}$  $\mathbf{k}_{s}^{H}=\left[ \begin{matrix} k_{s,1}^{H} & k_{s,2}^{H} \end{matrix} \right]=\left[ \begin{matrix} -1.888 & 9.52 \end{matrix} \right]a^{-1}$ (in $\mathbf{x}^{'}$-coordinate)  $\mathbf{B}^{H}=p_{s}B_{=}^{H}{\hat{\mathbf{a}}}_{=}\boldsymbol{+}p_{s}B_{\parallel}^{H}{\hat{\mathbf{a}}}_{\parallel}$  $=-1.18\times{10}^{-3}a^{-2}{\hat{\mathbf{a}}}_{=}-6.7844\times{10}^{-3}a^{-2}{\hat{\mathbf{a}}}_{\parallel}$  $=\left[ \begin{matrix} -0.83438 & -6.7844 & 0.83438 \end{matrix} \right]\times{10}^{-3}a^{-2}$  $=\left[ \begin{matrix} -0.94399 & -7.6756 & 0.94399 \end{matrix} \right]p_{s}a^{-1}$ (in $\mathbf{x}$-coordinate)  $B^{H}=6.8862\times{10}^{-3}a^{-2}=7.7909p_{s}a^{-1}$  $l_{H}=12.0506a$  $l_{H}^{-1}=0.082983a^{-1}$ |
| --- |


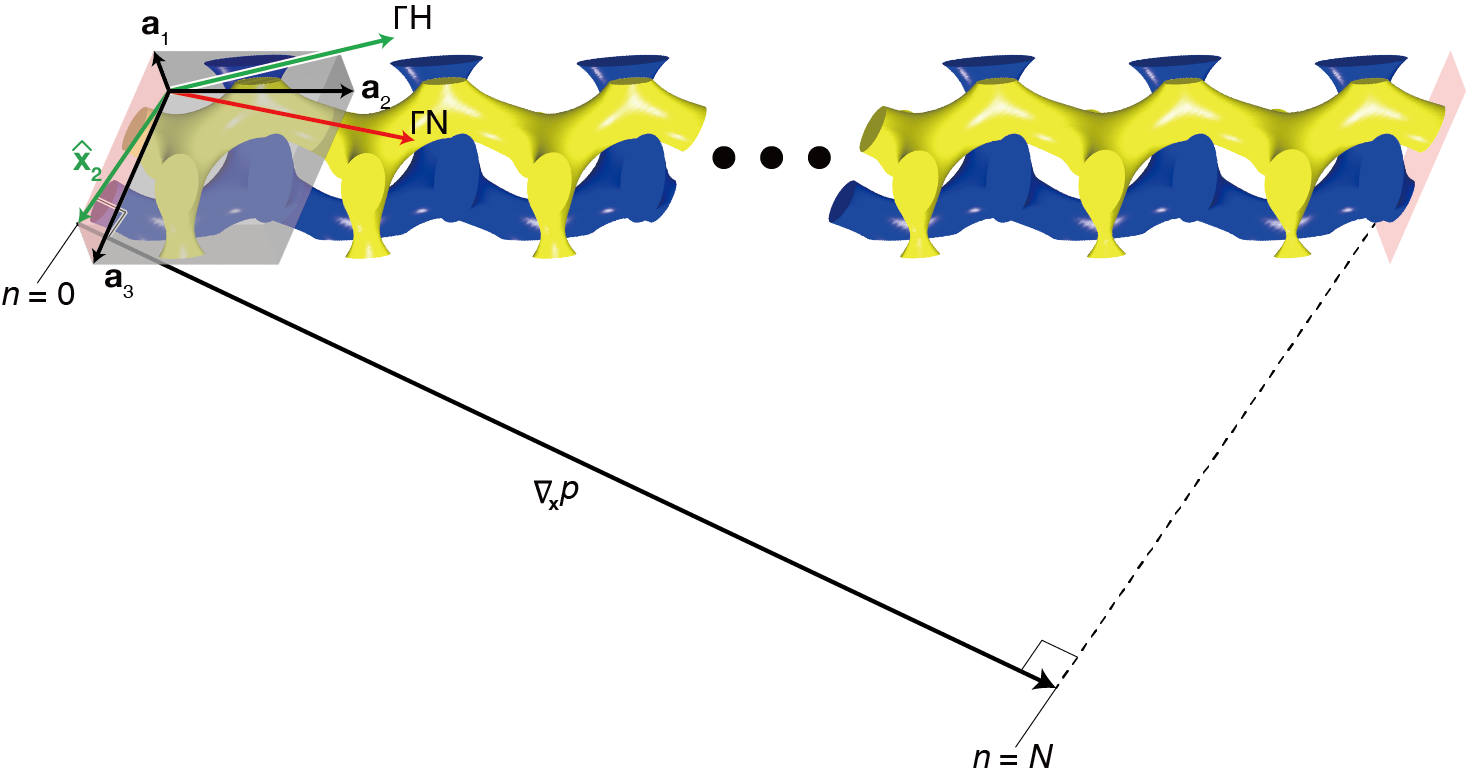


**Fig. S6. DG primitive cells array that was input to get Fig. 4-6 in the main text.** The array consists of $N$ cells with spatially linearly changing $p$ along ${\hat{\mathbf{a}}}_{\perp}$-direction. Several directions significantly dealt with in this study are also marked.

**5. Pseudomagnetic field with a nonlinear perturbation**

It is worth to think of a situation when Equation S12 or ${\hat{\mathbf{k}}}_{s}\delta k=\mathbf{A}$ is nonlinear. If we suppose that the nonlinear perturbation’s mathematical formula is infinitely differentiable relation, we can impose a nonlinear polynomial onto one of Equation S12 or ${\hat{\mathbf{k}}}_{s}\delta k=\mathbf{A}$. Whatever equation we select, Equation S18 is modified as follows:

|  | $\mathbf{A}^{\mathbf{'}}=\left[ k_{s,1}\left\{ p\left( \mathbf{x}^{\mathbf{'}} \right)-p_{0} \right\}^{m},k_{s,2}\left\{ p\left( \mathbf{x}^{\mathbf{'}} \right)-p_{0} \right\}^{m},0 \right]=\left( \frac{p_{s}}{\sqrt{2}} \right)^{m}\left\{ \frac{1}{\sqrt{2}}\left( x_{1}^{'}-x_{3}^{'} \right)+x_{2}^{'}-\sqrt{2}d_{0} \right\}^{m}\left[ k_{s,1},k_{s,2},0 \right] ,$ | (S23) |
| --- | --- | --- |

and we have

|  | $\mathbf{B}^{\mathbf{'}}=\nabla_{\mathbf{x}^{'}}\times\mathbf{A}^{\mathbf{'}}$ |  |
| --- | --- | --- |
|  | $=\left( \frac{m}{\sqrt{2}} \right)\left( \frac{p_{s}}{\sqrt{2}} \right)^{m}\left\{ \frac{1}{\sqrt{2}}\left( x_{1}^{'}-x_{3}^{'} \right)+x_{2}^{'}-\sqrt{2}d_{0} \right\}^{m-1}\left[ k_{s,2},-k_{s,1},k_{s,2}-\sqrt{2}k_{s,1} \right]$ |  |
|  | $=m\left( \frac{p_{s}}{\sqrt{2}} \right)^{m}\left\{ \frac{1}{\sqrt{2}}\left( x_{1}^{'}-x_{3}^{'} \right)+x_{2}^{'}-\sqrt{2}d_{0} \right\}^{m-1}\left[ k_{s,1}\frac{\left[ 1,-\sqrt{2},-1 \right]}{2} \right.$ |  |
|  | $\left. -\left( k_{s,2}-\frac{k_{s,1}}{\sqrt{2}} \right)\frac{\left[ -1,0,-1 \right]}{\sqrt{2}} \right].$ |  |
|  |  | (S24) |

The above pseudomagnetic field with respect to the original coordinate system is written as follows:

|  | $\mathbf{B}=mp_{s}\left\{ \frac{p_{s}}{\sqrt{2}}\left( x_{1}+x_{3} \right) \right\}^{m-1}\frac{1}{\sqrt{2}}\left[ k_{s,1}\frac{\left[ 1,0,-1 \right]}{\sqrt{2}}-\left( k_{s,2}-\frac{k_{s,1}}{\sqrt{2}} \right)\left[ 0,1,0 \right] \right]$ |  |
| --- | --- | --- |
|  | $=mp_{s}\left\{ \frac{p_{s}}{\sqrt{2}}\left( x_{1}+x_{3} \right) \right\}^{m-1}\left( B_{=}{\hat{\mathbf{a}}}_{=}\boldsymbol{+}B_{\parallel}{\hat{\mathbf{a}}}_{\parallel} \right)$ | (S25) |

where $B_{=}$ and $B_{\parallel}$ are given by Equations S21 and S22, respectively, and ${\hat{\mathbf{a}}}_{=}\boldsymbol{=}\left[ 1,0,-1 \right]/\sqrt{2}$ and ${\hat{\mathbf{a}}}_{\parallel}\boldsymbol{=}\left[ 0,1,0 \right]$ are the unit vectors of $\mathbf{a}_{=}=-\mathbf{a}_{1}+\mathbf{a}_{3}$ and $\mathbf{a}_{\parallel}=\mathbf{a}_{1}+\mathbf{a}_{3}$, respectively, placed on the boundary (see Fig. 4a in the main text). Compared to Equation S20, the direction remains and only the magnitude becomes position-dependent by the multiplication of $m\left\{ {p_{s}\left( x_{1}+x_{3} \right)}/\sqrt{2} \right\}^{m-1}$.

As an example, we investigate the photonic behavior of a supercell whose perturbation along the $\mathbf{a}_{\perp}$-direction (see Fig. S7a) is given by

|  | $p\left( \mathbf{x} \right)=\left( \frac{1}{d_{0}} \right)^{3}\frac{p_{Max}-p_{Min}}{2}\left\{ d\left( \mathbf{x} \right)-d_{0} \right\}^{3}+p_{0}$ | (S26) |
| --- | --- | --- |

where $d_{0}$ is a distance between the planes at $n=0$ and $n=N/2$, and the constant $p_{0}=\left( p_{Max}+p_{Min} \right)/2$ is a central value of $p\left( \mathbf{x} \right)$. The results shown in Fig. S7b-c have no significant difference from the results in Fig. S1b-c.

**6. Remarks on experimental realization**

Although we also have a plan for the experimental study, there are several hurdles so that it requires much time. First, our DG’s refractive index is 4.0, and this high value can be obtained if germanium^12^ or ceramic-filled plastic (e.g., C-STOCK AK)^13^ are used. Second, how to build the complex structure may be an issue if we use the material with the high-refractive index. Although there exist several methods, such as cutting a bulk structure^13^, 3D printing^14^, direct laser writing^15^, interference-lithography^6^, or self-assembly^16^, any method does not seem to be practical. Furthermore, the DG’s perturbation strength depends on the location. The structural difference between two unit cells around the opposite boundaries are not so big. We wonder if the several methods in the above can manufacture the difference accurately.

However, all the above discussion does not mean the ‘impossibility’ but ‘large duration’ of the experimental work. Nowadays, techniques and skills of building a complex 3D geometry become more accurate. The number of applicable materials is also growing. Therefore, we will report the experimental results as a separated report when we carry out the experiment.


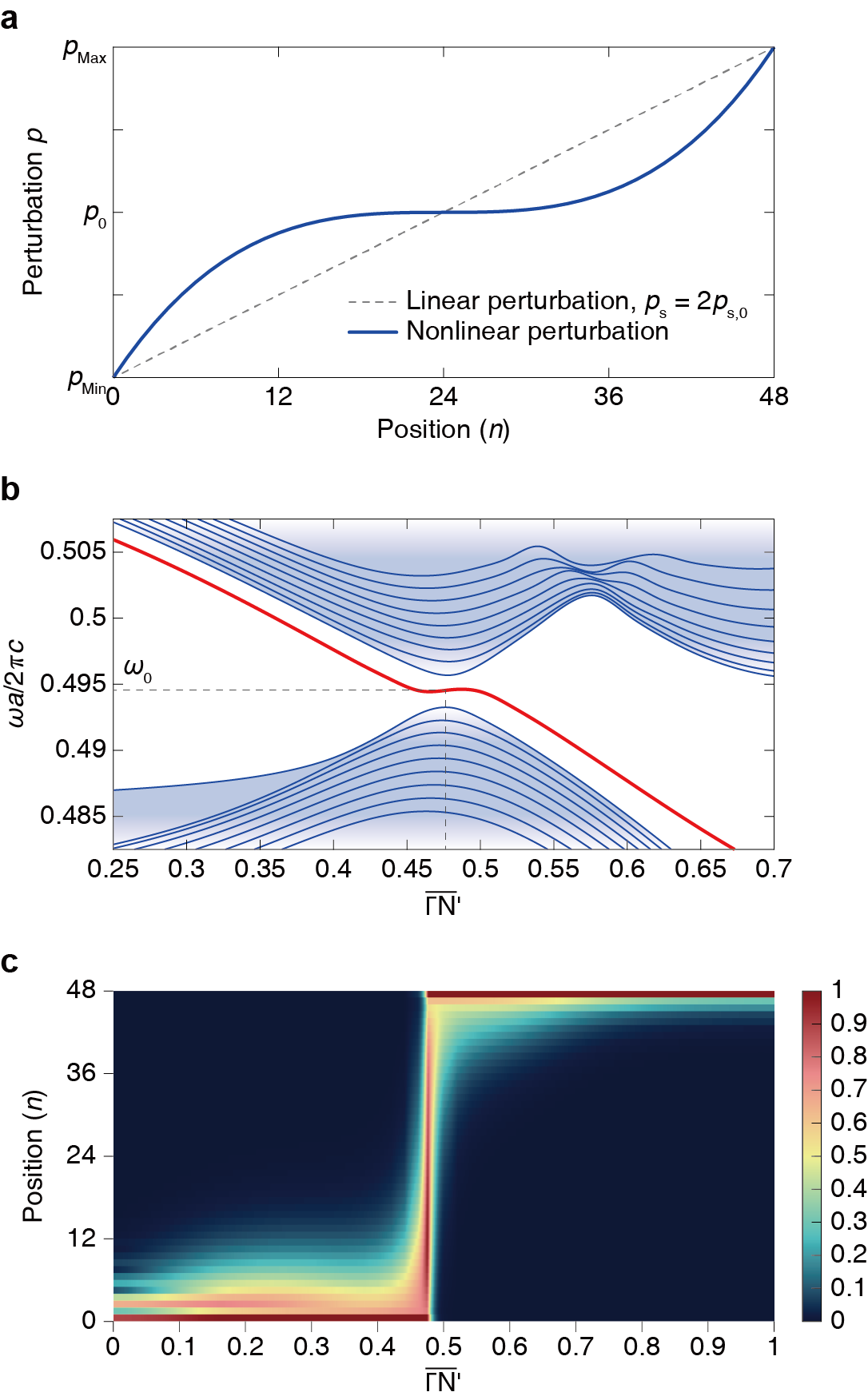


**Fig. S7. Photonic Landau levels and eigenstates in a pseudomagnetic field by a nonlinear spatial gradient of** $p$**.** **a**, Nonlinear perturbations (blue curve) along the $\mathbf{a}_{\perp}$-direction when $N=48$ is are used. The gray dotted line is the linear perturbation for comparisons between the nonlinear and linear perturbations **b**, Photonic band structures along $\Gamma N^{'}$-direction which exhibits Landau plateau at around $N_{0}$. **c**, Normalized eigenstates along the zeroth Landau level (red curve in **b**, respectively) to see surface states localized on the boundary. **b** and **c** are simulation results.

**7. Additional data about the evasion behavior of photonic waves**

Regarding the evasion behavior of photonic waves using heterogeneous blocks (shown in Fig. 7 in the main text), we investigate the effect of incident locations of photoniwaves on the evasion behavior. In Fig. S8a (or Fig. 7d-g in the main text), the reason of setting the star symbol around $n=0$ is that the blocks $m=\left[ 0, 8 \right]$ localizes the photonic wave around $n=0$. In other words, the blocks apart from the localization boundary suppress the wave propagation. If we set the star symbols in other locations as shown in Fig. S8b-d, the incident waves should pass through the wave-suppressing-blocks, thus the intensity exhibiting the evasion behavior becomes weaker.

We also perform the same simulation in Fig. 7g in the main text with different frequency inputs. Among the six frequencies marked in Fig. S9a-b, only the lowest two frequencies $\omega_{1}$ and $\omega_{2}$ are below the frequency of the zeroth Landau level’s plateau of the blocks $p_{s}=5p_{s,0}$, as shown in Fig. S9b. Thus, the wave localization properties at these frequencies of the Landau level are simple, and we get the clear evasion behavior as shown in Fig. S9c-d. Meanwhile $\omega_{3}$ and $\omega_{4}$ are in the plateau region of the zeroth Landau level, as shown in Fig. S9b. The localization of the wave in the blocks $m=\left[ 8, 24 \right]$ cannot be clearly determined, and the results becomes Fig. S9e-f. The higher frequencies $\omega_{5}$ and $\omega_{6}$ correspond to opposite localization in the blocks $p_{s}=-2p_{s,0}$, they are higher than the frequency of the zeroth Landau level’s plateau, as depicted in Fig. S9a. Thus, the incident waves cannot smoothly pass through the blocks $m=\left[ 0, 8 \right]$, as shown in Fig. S9g-h.


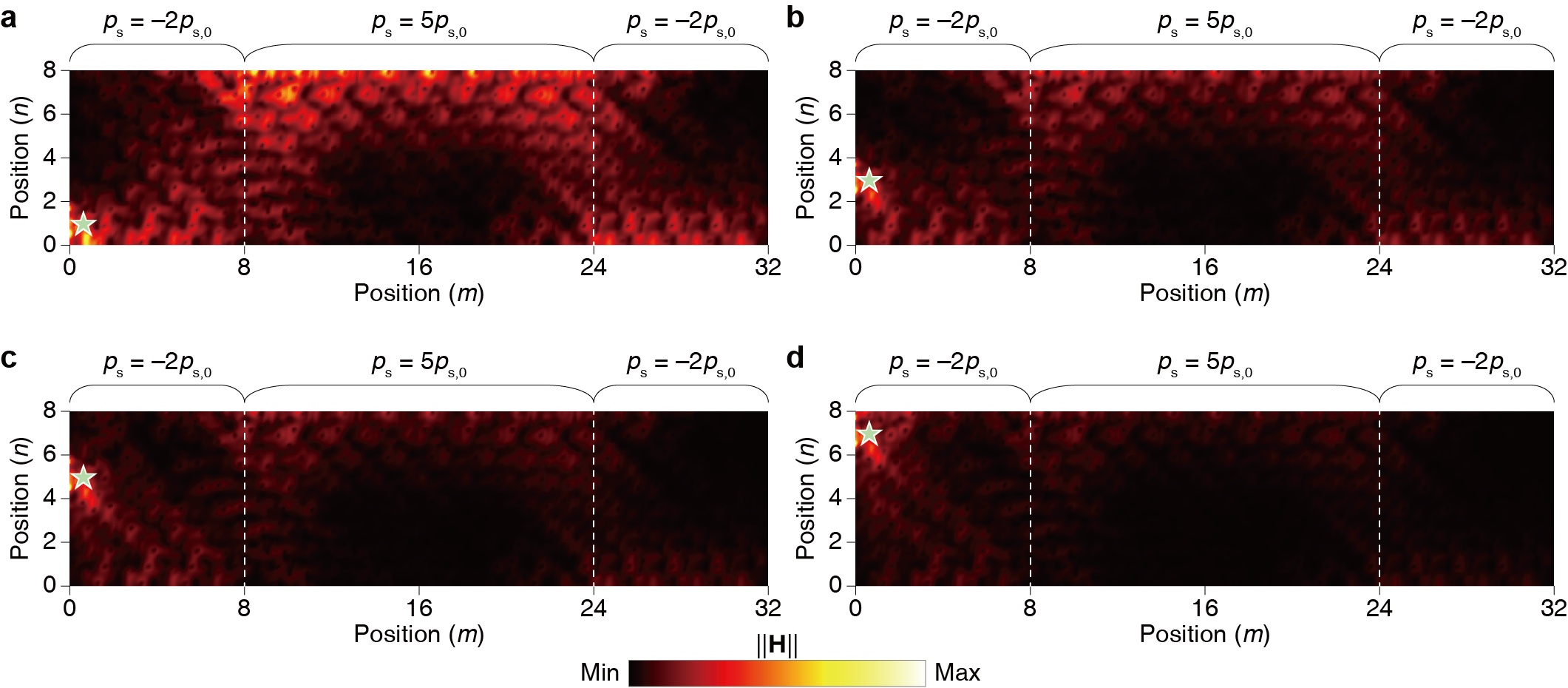


**Fig. S8. Evasion behavior of photonic waves with different incident locations.** Incident points are marked as the star symbols. Here, **a** is equivalent to Fig. 7g in the main text.

**8. Effective surface potential regarding a double gyroid array**

Although Fig. 1d and 8 plot $V_{s}$ the scalar coefficient of the surface potential, it is natural having curiosity about the surface potential that describes the photonic behaviors in Fig. 5 and 6 in the main text. Here, we set effective surface potentials for this purpose.

The amount of translation $h$ is related to the periodicity, e.g., $h=0.02$ and $h=1.02$ indicate the same structure when we replace $\mathbf{x}$ as $\mathbf{x}-h\mathbf{a}_{\perp}$ in the equation of the double gyroid structure. Thus, we set $V_{a}$, the maximum magnitude of $V_{s}$ (refer to Fig. S10a), as follows:

|  | $V_{a}=V_{a,0}\sin\left\{ 2\pi\left( h-h_{0} \right) \right\}+V_{a,1}$ | (S26) |
| --- | --- | --- |

Here, the parameters $V_{a,0}$, $V_{a,1}$, and $h_{0}$ need to be obtained empirically.

We re-calculate Fig. 1e in the main text and Fig. S4f with several $h$ through the band structure in Fig. S10b. The results in Fig. S10c-d exhibit the similar trend as Fig. 6c in the main text.


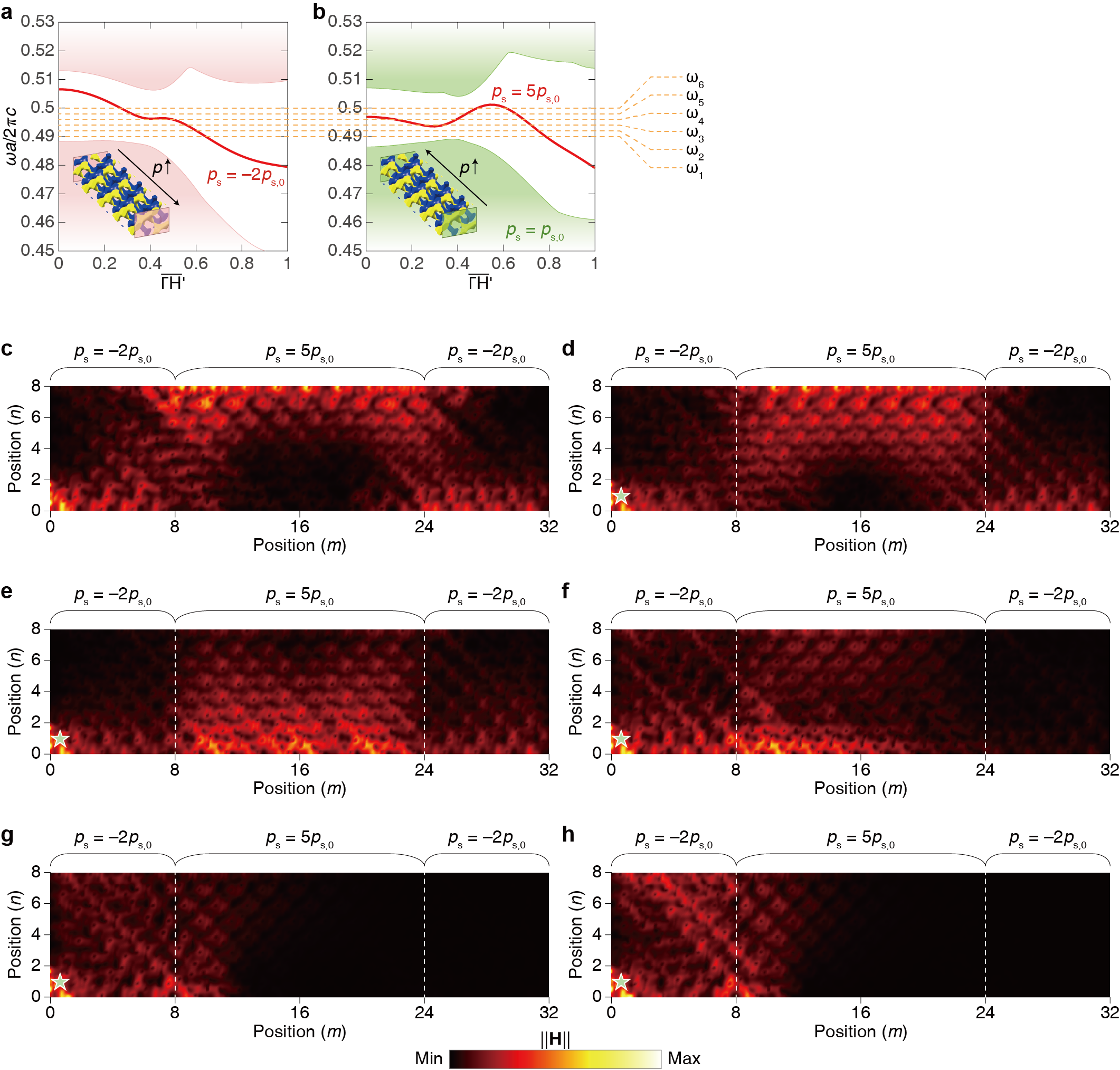


**Fig. S9. Photonic wave propagations with different input frequencies.** **a-b**, Zeroth Landau levels by the 8-block system with several perturbation fields, where $p_{s,0}=7.0711\times{10}^{-3}a^{-1}$. The perturbation fields used in **a** and **b** are opposite. The input frequencies used in the below panels are also marked. **c-h**, Simulated photonic wave propagations with the frequencies $\omega_{1}$ to $\omega_{6}$, respectively. Here, **c** is equivalent to Fig. 7g in the main text.


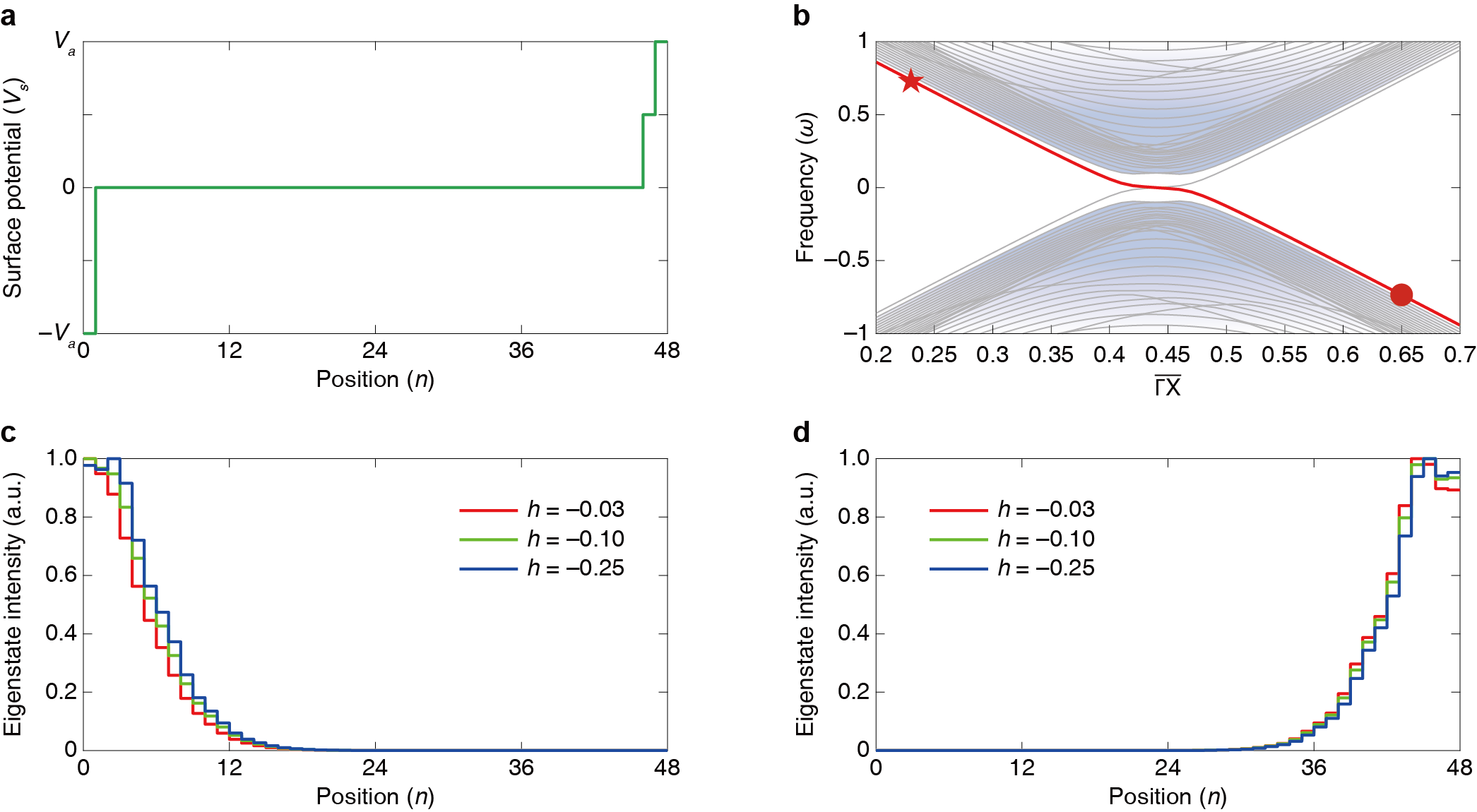


**Fig. S10. Adjustment of effective surface potential by** $V_{a}$ **of equation (S26) when Weyl equation in Section S2 is applied. a**, Plot of the surface potential’s scalar coefficient $V_{s}$. Its maximum and minimum values are given by $V_{a}$ and $-V_{a}$, respectively. **b**, Band structure when $V_{a}=0$ (i.e., $h=0$), which is the same as Fig. S3b. Only two symbols are denoted to mark specific points. **b-c**, Eigenstate intensities at the point marked by the star (**b**) and circle symbols (**c**) in **a**, respectively, for three $V_{a}$ tuned by translation $h$. In equation (S26), we use $h_{0}=0$ and $V_{a,0}=V_{a,1}=0.1a^{-1}$. All other components to calculate the band structures and eigenstates are the same as Section S2.

**References**

1 Potter, A. C., Kimchi, I. & Vishwanath, A. Quantum oscillations from surface Fermi arcs in Weyl and Dirac semimetals. *Nature Communications* **5**, 5161 (2014).

2 Bhowmick, S. & Shenoy, V. B. Weber-Fechner type nonlinear behavior in zigzag edge graphene nanoribbons. *Physical Review B* **82**, 155448 (2010).

3 Shtanko, O. & Levitov, L. Robustness and universality of surface states in Dirac materials. *Proceedings of the National Academy of Sciences* **115**, 5908-5913 (2018).

4 Dou, Z. et al. Imaging Bulk and Edge Transport near the Dirac Point in Graphene Moiré Superlattices. *Nano Letters* **18**, 2530-2537 (2018).

5 Dongre, N. K. & Roychowdhury, K. Effects of surface potentials on Goos-Haenchen and Imbert-Fedorov shifts in Weyl semimetals. *arXiv preprint arXiv:2106.04573* (2021).

6 Park, H. & Lee, S. Double Gyroids for Frequency-Isolated Weyl Points in the Visible Regime and Interference Lithographic Design. *ACS Photonics* **7**, 1577-1585 (2020).

7 Yu, R., Qi, X. L., Bernevig, A., Fang, Z. & Dai, X. Equivalent expression of Z2 topological invariant for band insulators using the non-Abelian Berry connection. *Physical Review B* **84**, 075119 (2011).

8 Wang, Q., Xiao, M., Liu, H., Zhu, S. & Chan, C. T. Optical Interface States Protected by Synthetic Weyl Points. *Physical Review X* **7**, 031032 (2017).

9 Soluyanov, A. A. et al. Type-II Weyl semimetals. *Nature* **527**, 495 (2015).

10 Yang, Z. et al. Weyl points in a magnetic tetrahedral photonic crystal. *Opt. Express* **25**, 15772-15777 (2017).

11 Jia, H. et al. Observation of chiral zero mode in inhomogeneous three-dimensional Weyl metamaterials. *Science* **363**, 148-151 (2019).

12 Lu, L., Fu, L., Joannopoulos, J. D. & Soljačić, M. Weyl points and line nodes in gyroid photonic crystals. *Nature Photonics* **7**, 294 (2013).

13 Lu, L. et al. Experimental observation of Weyl points. *Science* **349**, 622-624 (2015).

14 Pouya, C. et al. Characterization of a Mechanically Tunable Gyroid Photonic Crystal Inspired by the Butterfly Parides Sesostris. *Advanced Optical Materials* **4**, 99-105 (2016).

15 Goi, E., Yue, Z., Cumming, B. P. & Gu, M. Observation of Type I Photonic Weyl Points in Optical Frequencies. *Laser & Photonics Reviews* **12**, 1700271 (2018).

16 Fruchart, M. et al. Soft self-assembly of Weyl materials for light and sound. *Proceedings of the National Academy of Sciences of the United States of America* **115**, E3655-E3664 (2018).
